# Nanobody-based recombinant antivenom for cobra, mamba and rinkhals bites

**DOI:** 10.1038/s41586-025-09661-0

**Published:** 2025-10-29

**Authors:** Shirin Ahmadi, Nick J. Burlet, Melisa Benard-Valle, Alid Guadarrama-Martínez, Samuel Kerwin, Iara A. Cardoso, Amy E. Marriott, Rebecca J. Edge, Edouard Crittenden, Edgar Neri-Castro, Monica L. Fernandez-Quintero, Giang T. T. Nguyen, Carol O’Brien, Yessica Wouters, Konstantinos Kalogeropoulos, Suthimon Thumtecho, Tasja Wainani Ebersole, Camilla Holst Dahl, Emily U. Glegg-Sørensen, Tom Jansen, Kim Boddum, Evangelia Manousaki, Esperanza Rivera-de-Torre, Andrew B. Ward, J. Preben Morth, Alejandro Alagón, Stephen P. Mackessy, Stuart Ainsworth, Stefanie K. Menzies, Nicholas R. Casewell, Timothy P. Jenkins, Anne Ljungars, Andreas H. Laustsen

**Affiliations:** 1 Department of Biotechnology and Biomedicine, Technical University of Denmark, Kongens Lyngby, Denmark; 2 Departamento de Medicina Molecular y Bioprocesos, Instituto de Biotecnología, Universidad Nacional Autónoma de México, Cuernavaca, México; 3 Department of Biological Sciences, University of Northern Colorado, Greeley, CO USA; 4 Centre for Snakebite Research and Interventions, Department of Tropical Disease Biology, Liverpool School of Tropical Medicine, Liverpool, UK; 5 School of Biochemistry, Biomedical Sciences Building, University of Bristol, Bristol, UK; 6 Department of Infection Biology and Microbiomes, Institute of Infection, Veterinary and Ecological Sciences, University of Liverpool, Liverpool, UK; 7 Department of Integrative Structural and Computational Biology, The Scripps Research Institute, La Jolla, CA USA; 8 Division of Toxicology, Department of Medicine, Chulalongkorn University and King Chulalongkorn Memorial Hospital, the Thai Red Cross Society, Bangkok, Thailand; 9 Sophion Bioscience, Ballerup, Denmark; 10 Biomedical and Life Sciences, Faculty of Health and Medicine, Lancaster University, Lancaster, UK

**Keywords:** Antibody therapy, Preclinical research

## Abstract

Each year, snakebite envenoming claims thousands of lives and causes severe injury to victims across sub-Saharan Africa, many of whom depend on antivenoms derived from animal plasma as their sole treatment option^1^. Traditional antivenoms are expensive, can cause adverse immunological reactions, offer limited efficacy against local tissue damage and are often ineffective against all medically relevant snake species^2^. There is thus an urgent unmet medical need for innovation in snakebite envenoming therapy. However, developing broad-spectrum treatments is highly challenging owing to the vast diversity of venomous snakes and the complex and variable composition of their venoms^3^. Here we addressed this challenge by immunizing an alpaca and a llama with the venoms of 18 different snakes, including mambas, cobras and a rinkhals, constructing phage display libraries, and identifying high-affinity broadly neutralizing nanobodies. We combined eight of these nanobodies into a defined oligoclonal mixture, resulting in an experimental polyvalent recombinant antivenom that was capable of neutralizing seven toxin families or subfamilies. This antivenom effectively prevented venom-induced lethality in vivo across 17 African elapid snake species and markedly reduced venom-induced dermonecrosis for all tested cytotoxic venoms. The recombinant antivenom performed better than a currently used plasma-derived antivenom and therefore shows considerable promise for comprehensive, continent-wide protection against snakebites by all medically relevant African elapids.

## Abstract (web summary)

A recombinant antivenom composed of eight nanobodies provides broad protection against venom-induced lethality and dermonecrosis in mice challenged with venoms from cobras, mambas and rinkhals snakes.

## Main

**Fig. 1:**
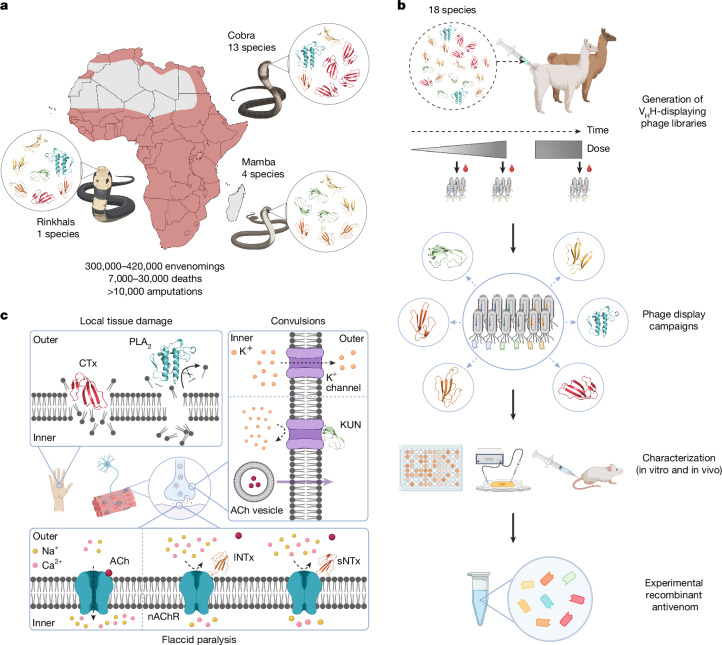
Schematic overview of the study and the function of targeted toxin families. **a**, Distribution of the 18 most medically relevant elapid snakes from sub-Saharan Africa with the estimated snakebite envenoming incidence, fatality rate and morbidity^1,61^. **b**, Schematic overview of the pipeline used for the development of the experimental recombinant antivenom. An alpaca and a llama were immunized with a mixture of whole venoms from 18 elapid snakes to generate immune V_H_H-displaying phage libraries for discovery of V_H_Hs with broad cross-reactivity and high affinity. The V_H_Hs were characterized in vitro and in vivo, and eight V_H_Hs were chosen and combined into an experimental recombinant antivenom. **c**, Visual representation of the pathology of the included major toxin families. A type IA cytotoxin^62^ (CTx, dark red, Protein Data Bank (PDB) ID: 9BK6) disrupts lipid bilayers, and a PLA_2_ (cyan, PDB ID: 1A3D)^63^ catalyses the hydrolysis of fatty acids from phospholipids, both of which cause local tissue damage^37,64,65^. An sNTx^66^ (dark orange, PDB ID: 1VB0) and an lNTx^62^ (light orange, PDB ID: 9BK5) both block acetylcholine (ACh) from binding to nAChRs, preventing ion flux and leading to flaccid paralysis^31^. A KUN^67^ (light green, PDB ID: 1DEM) blocks voltage-gated K^+^ channels, causing enhanced acetylcholine release and subsequent convulsions^68^. Created in BioRender. Burlet, N. (2025) https://BioRender.com/1lypaqd.

Snakebite envenoming is a neglected tropical disease that disproportionately affects populations in rural tropical regions, with sub-Saharan Africa bearing a substantial burden. It is estimated that more than 300,000 snakebites occur annually in this region, leading to over 7,000 deaths and 10,000 amputations, although the actual incidence and mortality may be up to 5 times higher^1,4^ (Fig. 1a). Currently, the only specific treatment for snakebite envenoming is antivenoms derived from the plasma of hyperimmunized large animals, such as horses. Although they are life-saving, these antivenoms suffer from batch-to-batch variation, high production costs, sometimes restricted efficacy across species (that is, limited polyvalency), and the risk of causing immunological adverse reactions^2,5,6^. In addition, existing antivenoms may contain as little as 10% active ingredient—that is, antibodies specifically neutralizing venom toxins^7^. Consequently, large doses need to be administered, increasing treatment costs. Furthermore, current antivenoms are often ineffective at mitigating local tissue damage caused by envenoming, such as dermonecrosis, which contributes to the high number of amputations and tissue debridement procedures among snakebite victims^8–10^.

To overcome the limitations of current antivenoms, the development of alternative broad-spectrum antivenoms, with a high content of therapeutically active antibodies and improved efficacy against both lethality and local tissue damage, is critically warranted. To this end, recombinant monoclonal broadly neutralizing antibodies have shown great promise^11^. Specifically, it has been demonstrated that a single broadly neutralizing antibody targeting a specific toxin (sub)family can prevent toxicity and/or lethality in mice injected with whole venoms predominantly composed of toxins from that same (sub)family^12–17^. In addition, mixtures of a few recombinant monoclonal antibodies or antigen-binding fragments of camelid heavy-chain-only antibodies, V_H_Hs (nanobodies), have been shown to neutralize at least one or two venoms^18–21^. However, to truly qualify as an alternative to existing therapy, a recombinant antivenom must effectively neutralize venoms from all medically relevant snake species within a region, across different genera. This is particularly challenging to achieve owing to the complex compositions of snake venoms, where the venom from a single species can contain up to a hundred toxins from multiple different protein families^22^, which can vary extensively both inter- and intraspecifically^3^. This complexity has created a prevailing view that neutralizing all medically relevant toxins will require an impractically large number of antibodies.

Here we demonstrate that neutralization of 17 out of the 18 most medically relevant elapid snakes in sub-Saharan Africa can be achieved with a surprisingly low number of broadly neutralizing toxin-targeting V_H_Hs. After identifying the medically relevant toxins in the venoms of the 18 elapid snakes (mambas, cobras and rinkhals), we used phage display technology^23,24^ to identify V_H_Hs that were evaluated for their ability to bind and neutralize their target toxins (Fig. 1b). We then designed an experimental recombinant antivenom based on an oligoclonal mixture of the top 8 V_H_Hs and demonstrated that this V_H_H cocktail can preclinically prevent venom-induced lethality against all of the 18 most medically relevant elapid snakes in sub-Saharan Africa, except *Dendroaspis angusticeps*, in pre-incubation experiments. We further validated the efficacy of the antivenom using a representative panel of 11 venoms in a more stringent rescue model that better simulates a real-world snakebite scenario. We also demonstrated that the recombinant antivenom can inhibit or reduce morbidity-causing venom-induced dermonecrosis, a previously unexplored therapeutic area for V_H_Hs. Collectively, this study demonstrates the feasibility of developing a polyvalent recombinant antivenom with continent-wide species coverage, offering hope for advancing such snakebite envenoming therapeutics to future clinical application.

## Discovery of V_H_Hs against elapid toxins

Africa is inhabited by many venomous snake species, primarily belonging to the viperid or elapid families; however, many are of no or little medical concern. As outlined by the World Health Organization (WHO), within sub-Saharan Africa there are a total of 18 elapid snakes that can be considered the most medically important^25,26^ (Fig. 1a and Supplementary Table 1). They belong to 3 genera: *Dendroaspis* (mambas; 4 species), *Hemachatus* (rinkhals; 1 species) and *Naja* (cobras; 13 species); with the genus *Naja* being further divided into 3 subgenera: *Uraeus* (cape cobras; 5 species), *Boulengerina* (forest/water cobras; 1 species) and *Afronaja* (African spitting cobras; 7 species). The clinical manifestations of envenoming vary markedly among these five (sub)genera. Species belonging to *Dendroaspis*,* Uraeus* and *Boulengerina* primarily induce neurotoxic symptoms, whereas species belonging to *Hemachatus* and *Afronaja* mostly cause severe local tissue damage^27^ (Fig. 1c). Neutralizing the wide range of toxic effects that underlie these manifestations is thus a complex endeavour, which complicates the development of effective therapies with broad coverage of syndromes and snake species^28^.

To identify V_H_Hs targeting toxins from these venoms, we first generated immune V_H_H-displaying phage libraries from an alpaca and a llama injected with a mixture of venoms from the 18 medically important elapid snakes (Fig. 1b). Injection doses were increased across bi-weekly intervals over 8 time points, and 3 additional booster injections were administered 52, 54 and 60 weeks after the first immunization^20^. We collected blood samples at different time points during the immunization time course to generate three unique V_H_H-displaying phage libraries (Supplementary Table 2).

**Extended Data Fig. 1:**
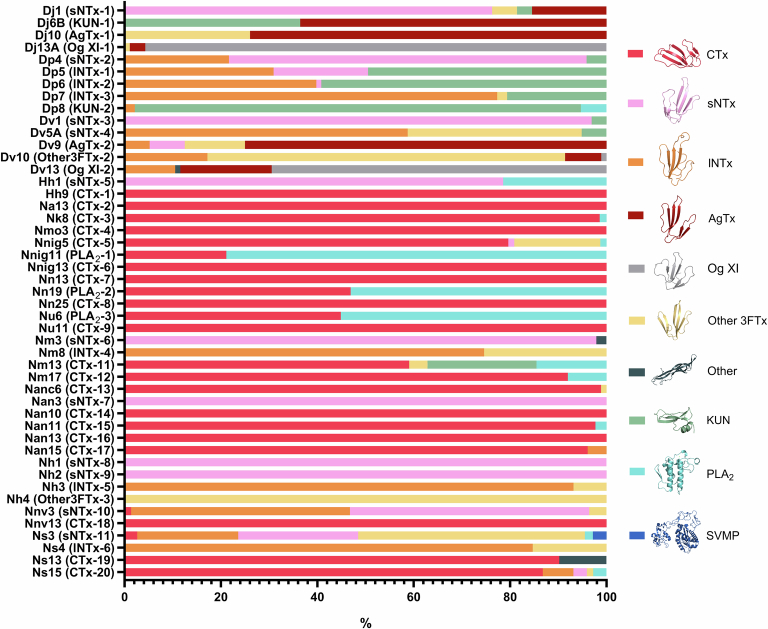
Proteomics of venom fractions. The abundance of different toxin (sub)families in the venom fractions obtained through RP-HPLC and analyzed with LC-MS/MS. Abbreviations can be found in Supplementary Table 1. The composition of the whole venoms can be found in an earlier study by Nguyen et al.^29^.

We next identified the most medically relevant toxins present in the 18 elapid venoms, on the basis of our previous work^29^. These toxins belong to three distinct protein families: three-finger toxins (3FTx), phospholipase A_2_ (PLA_2_) and Kunitz-type serine protease inhibitors (KUN). We purified the toxins using reversed-phase high-performance liquid chromatography (RP-HPLC) and performed proteomic analysis of the 47 fractions in which the key toxins were expected to be present (Extended Data Fig. 1 and Supplementary Table 1). The most abundant 3FTx found in these fractions belonged to five different subfamilies, namely type I α-neurotoxin (sNTx), type II α-neurotoxin (lNTx), type IA/IB cytotoxin (CTx), orphan group XI (Og XI) and aminergic toxin (AgTx)^30–32^. In this study, fractions and purified toxins are denoted by the name of the main medically relevant toxin (sub)family present in the fraction, followed by a number (for example, lNTx-1, lNTx-2, and so on) for ease of reference (Supplementary Table 1).

**Extended Data Fig. 2:**
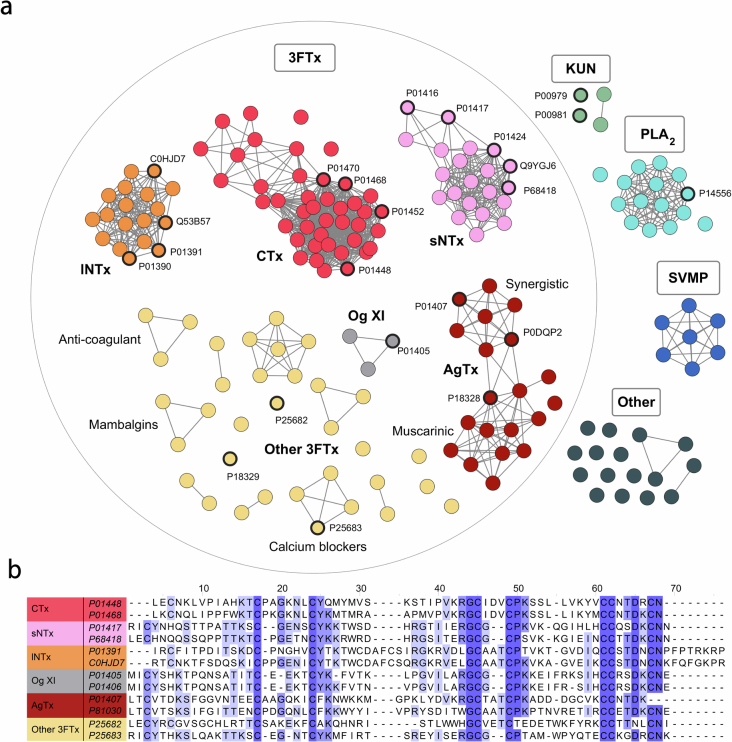
Clustering of toxins. **a**, Sequence similarity network (SSN) of the toxins present in the venoms of the 18 elapid snake species. Every circle represents a single toxin and a connecting line between 2 circles indicates a sequence similarity of at least 70%. The medically most relevant toxins within the fractions used as target antigens in the phage display selection campaigns are outlined with a bold border and annotated with the Uniprot accession number of the exact toxin or its closest known homolog. **b**, A sequence alignment of representative toxins (or closest known homolog) from each of the 3FTx sub-families, illustrating the large diversity between these toxins.

Overall, 16 fractions (3 lNTx, 3 sNTx, 2 KUN, 2 Og XI, 1 AgTx, 1 PLA_2_ and 4 CTx) were selected to be used as targets in subsequent phage display selections, on the basis of their abundance in the corresponding venom and their purity (Supplementary Table 3, Supplementary Table 1, Extended Data Fig. 2). Throughout the phage display campaigns, we utilized cross-panning strategies^33,34^, including for example, exposure to sNTx fractions from different snake species and/or decreasing antigen concentrations in consecutive rounds to enrich for V_H_Hs with broad cross-reactivity and/or high affinity (Supplementary Table 3).

## V_H_Hs show broad neutralization in vitro

**Fig. 2:**
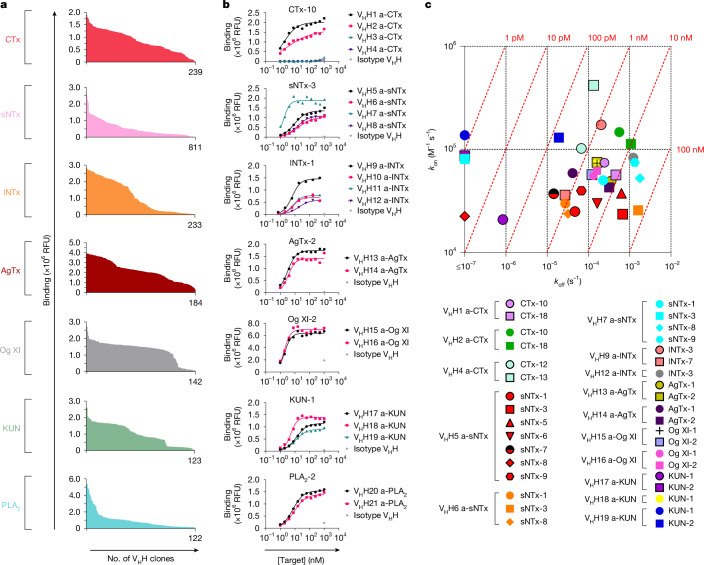
Screening, dose–response binding curves and isoaffinity plot of monoclonal V_H_Hs binding to toxins and venom fractions. **a**, Screening of more than 3,000 V_H_H clones for binding to their cognate toxin in an expression-normalized capture DELFIA. Only V_H_Hs with a signal intensity ten times above the background level are shown, and the number of clones exceeding this threshold is displayed on the *x* axis. RFU, relative fluorescence units. **b**, Dose-response binding curves for 21 V_H_Hs against a venom fraction or a toxin representing each targeted toxin (sub)family, measured using expression-normalized capture DELFIA. **c**, Isoaffinity plot of binding of 15 cross-reactive V_H_Hs to venom fractions and toxins from various snake (sub)genera measured with BLI. Owing to the instrument’s detection limit for *k*_off_ of 10^−7^ s^−1^, four points below this cutoff are plotted at ≤10^−7^ s^−1^. The red diagonal lines represent specific *K*_d_ values, which are labelled above each line. *k*_on_, association rate.

**Extended Data Fig. 3:**
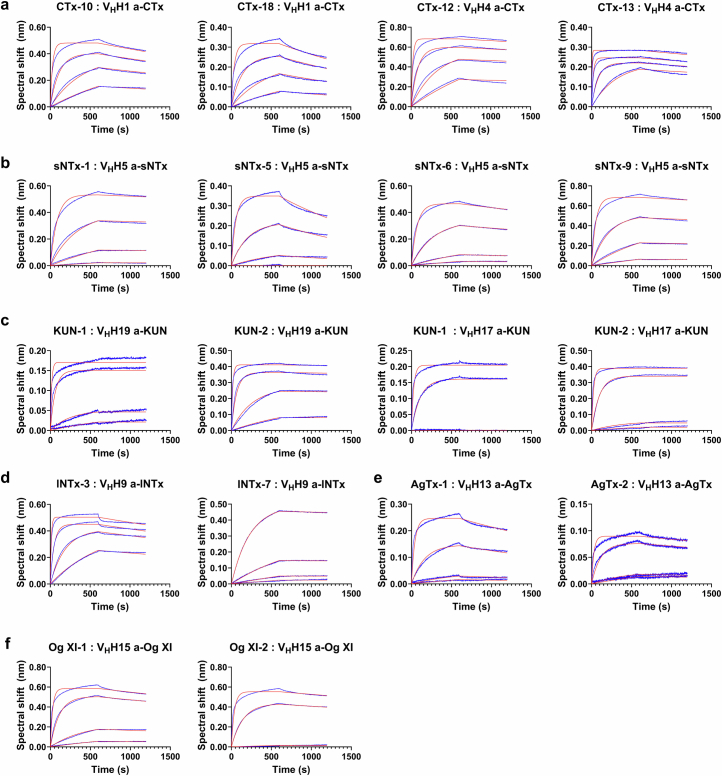
Sensorgrams of representative V_H_Hs showing association and dissociation to various venom fractions and toxins in BLI. **a**, V_H_H1 a-CTx binding to CTx-10 and CTx-18; V_H_H4 a-CTx binding to CTx-12 and CTx-13. **b**, V_H_H5 a-sNTx binding to sNTx-1, sNTx-5, sNTx-6, and sNTx-9. **c**, V_H_H19 a-KUN and V_H_H17 a-KUN binding to KUN-1 and KUN-2. **d**, V_H_H9 a-lNTx binding to lNTx-3 and lNTx-7. **e**, V_H_H13 a-AgTx binding to AgTx-1 and AgTx-2. **f**, V_H_H15 a-Og XI binding to Og XI-1 and Og XI-2.

Following the phage display campaigns, we subcloned 15 phage pool outputs, expressed more than 3,000 monoclonal V_H_Hs in *Escherichia coli*, and screened them for binding to their cognate target toxins using an expression-normalized capture dissociation-enhanced lanthanide fluorescence immunoassay (DELFIA). Approximately 60% of the V_H_Hs bound to their target toxins (Fig. 2a), and 25% of the clones with varying signal intensity were tested for cross-reactivity in a secondary DELFIA-based screening. Here we observed that more than 50% of the V_H_Hs bound multiple toxins from the same toxin (sub)family, which after sequencing revealed more than 100 unique V_H_H clones. Sequences of the lead V_H_Hs and complementarity-determining region 3 of hit V_H_Hs are provided in Supplementary Table 4. We speculate that the identification of multiple high-affinity, cross-reactive V_H_Hs may have been facilitated by our immunization strategy, which involved multiple venoms with a broad range of similar and dissimilar toxins (antigens). After our screening campaign, we further evaluated the top 21 unique cross-reactive V_H_Hs against their corresponding toxin families in a dose–response DELFIA, yielding half-maximal effective concentrations (EC_50_s) ranging from 1 nM to 15 nM (Fig. 2b). Subsequently, we selected the top 15 V_H_Hs on the basis of broad cross-reactivity and low EC_50_s, and evaluated their binding kinetics using biolayer interferometry (BLI). All of the 15 tested cross-reactive V_H_Hs displayed low nanomolar affinity (*K*_d_) with slow dissociation rates (*k*_off_ < 5.5 × 10^−4^ s^−1^) for most of their target toxins (Fig. 2c, Extended Data Fig. 3 and Supplementary Table 5).

**Fig. 3:**
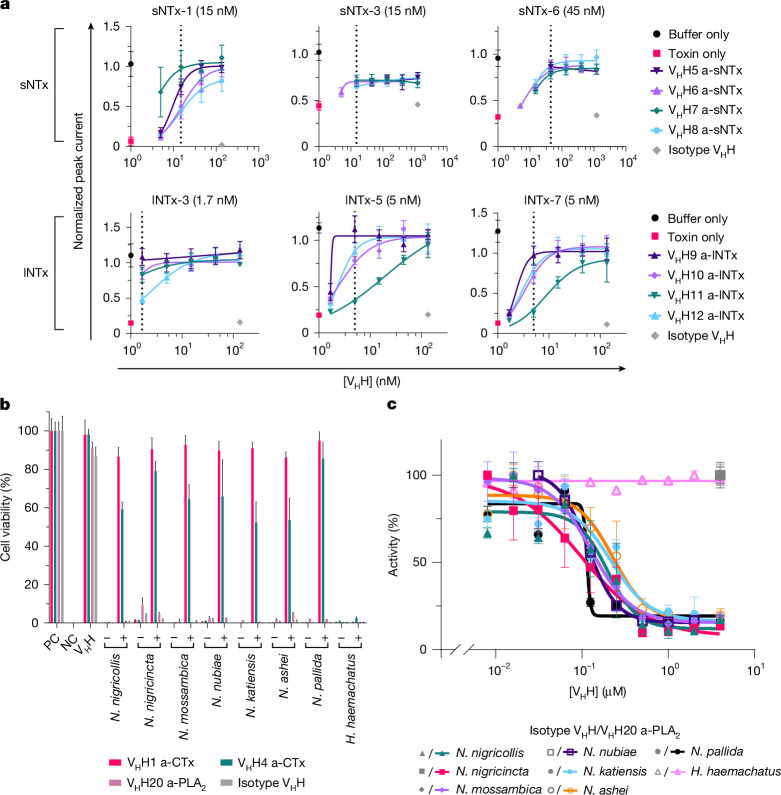
In vitro neutralization of sNTx, lNTx, CTx and PLA_2_. **a**, Neutralization of sNTx- and lNTx-mediated blocking of the muscle type nAChR current in whole-cell patch clamp. Dose-response curves are shown with increasing concentrations of V_H_H to prevent the blocking of nAChRs by sNTx-1, sNTx-3, sNTx-6, lNTx-3, lNTx-5 and lNTx-7. Experiments were performed using eight technical replicates, and results are expressed as mean ± s.d. Toxin concentrations are shown in parentheses, and the dotted line represents a 1:1 molar ratio of toxin:V_H_H. **b**, Neutralization of venom-induced cytotoxicity using a cell viability assay with a N/TERT keratinocyte cell line. The positive control (PC) includes medium supplemented with PBS and was set to 100% cell viability. For the negative control (NC), medium supplemented with Triton X-100 was added, resulting in complete cell death. As an additional control, V_H_H without any venom was included (V_H_H). For all venoms, − indicates 2 × half-maximal inhibitory concentration (IC_50_) of the venom without V_H_H addition, and + indicates venom incubated with a V_H_H at a 1:5 molar ratio of CTx or PLA_2_ to V_H_H. Experiments were conducted in triplicate, and results are expressed as mean ± s.d. **c**, Neutralization of PLA_2_ enzymatic activity in the whole venoms of *H. haemachatus* and all *Afronaja* species by V_H_H20 a-PLA_2_ using a colorimetric enzymatic activity assay with the chromogenic substrate 4-nitro-3-(octanoyloxy)benzoic acid (NOBA). Experiments were conducted in duplicate, and results are expressed as mean ± s.d.

Next, we evaluated the ability of these V_H_Hs to neutralize their target toxins in vitro. lNTx and sNTx exert their function by binding to the nicotinic acetylcholine receptors (nAChRs) at neuromuscular junctions, preventing acetylcholine binding and ion influx, which disrupts nerve–muscle communication and often results in paralysis^35^ (Fig. 1c). To address the neutralization of lNTx and sNTx, we measured nAChR currents in a human rhabdomyosarcoma cell line in patch clamp assays^36^ and observed that pre-incubation of anti-lNTx (a-lNTx) V_H_Hs with lNTx-3, lNTx-5 and lNTx-7 or anti-sNTx V_H_Hs with sNTx-1, sNTx-3 and sNTx-6 before addition to the cells protected the nAChR-mediated current—that is, they showed complete inhibition of neurotoxicity down to a 1:1 molar ratio between V_H_H and toxin (Fig. 3a).

Besides neurotoxicity, envenoming from 8 of the 18 elapid snakes included in this study—7 *Afronaja* and 1 *Hemachatus* species—can cause severe local tissue damage. This is primarily owing to the toxic actions of CTx and PLA_2_ present in their venoms, acting either alone or synergistically^37,38^ (Fig. 1c). We therefore assessed the ability of the anti-CTx V_H_Hs and an anti-PLA_2_ V_H_H to neutralize venom-induced cytotoxicity in cell viability assays. Specifically, we pre-incubated venoms from the eight snake species with three different V_H_Hs individually—V_H_H1 a-CTx, V_H_H4 a-CTx and V_H_H20 a-PLA_2_—before addition to a keratinocyte cell line. Both anti-CTx V_H_Hs demonstrated broad neutralization, providing 75–95% and 50–85% protection, respectively, against the cytotoxic effects from the venoms of all seven *Afronaja* species, whereas V_H_H20 a-PLA_2_ showed almost no neutralization of any of the tested venoms (Fig. 3b). We observed no neutralization of *Hemachatus* *haemachatus* venom, which was in accordance with the binding data, which showed that neither V_H_H1 a-CTx nor V_H_H4 a-CTx bound to CTx from this venom. Among the 18 elapid snakes studied, PLA_2_ exhibit the highest enzymatic activity in *Afronaja*, moderate activity in *Boulengerina* and *Hemachatus*, and negligible activity in *Uraeus* and *Dendroaspis*^29^. Thus, we also assessed the neutralizing effect of V_H_H20 a-PLA_2_ in an enzymatic assay and observed complete inhibition of PLA_2_ activity in the *Afronaja* venoms, but no neutralization of *H.* *haemachatus* venom (Fig. 3c).

## Design of a V_H_H cocktail

After demonstrating in vitro neutralization of several medically important toxin subfamilies by multiple V_H_Hs, we performed a series of WHO-recommended mouse pre-incubation experiments^39,40^ to examine whether these neutralizing effects would translate to in vivo protection. We first determined the median lethal dose (LD_50_) for venom fractions, toxins and whole venoms when administered intravenously (Supplementary Table 6), after which venoms were pre-incubated with either single or multiple V_H_Hs and administered to mice, with survival monitored for 24 h.

**Extended Data Fig. 4:**
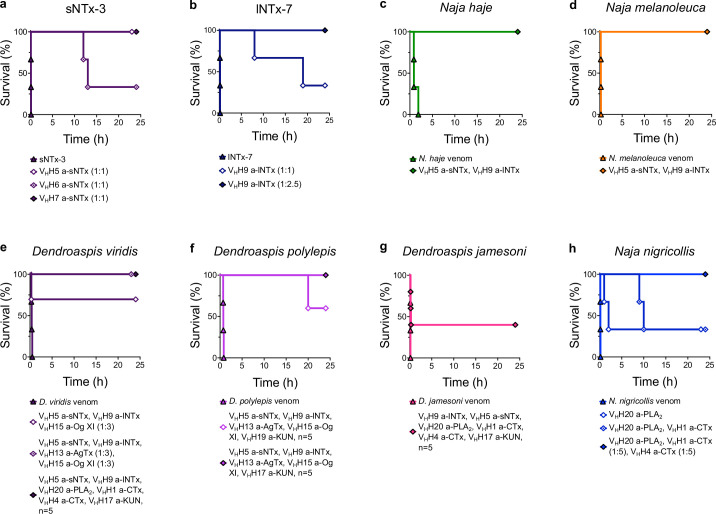
In vivo experiments to guide the design of a recombinant antivenom. Kaplan-Meier survival curves for mice challenged with sNTx-3 (**a**) lNTx-7 (**b**) or elapid snake venoms (**c** to **h**). 3 LD_50_s of each venom fraction, toxin, or venom were pre-incubated with either PBS or different mixtures of V_H_Hs and injected into mice using the i.v. route. n = 3 unless otherwise stated. Molar ratio between toxin (sub)family and the corresponding V_H_H is 1:10, unless otherwise specified in parentheses.

With the aim of having as few V_H_Hs as possible in the recombinant antivenom, we evaluated the most broadly neutralizing V_H_Hs against each toxin (sub)family. We started with neutralization of individual pure toxins or toxin fractions, then moved to simple whole venoms with a few different toxin subfamilies present, and finally evaluated the neutralization of more complex venoms. First, we evaluated the neutralization of sNTx-3 and lNTx-7 using V_H_H5 a-sNTx and V_H_H9 a-lNTx, respectively (Extended Data Fig. 4a,b and Supplementary Table 7); this resulted in the survival of all mice. Thereafter, we combined these two V_H_Hs and tested them on the venoms from *Naja haje* and *Naja melanoleuca*, species that are known to produce venoms rich in sNTx and lNTx. Pre-incubation of the venoms with these two V_H_Hs prevented lethality in all mice (Extended Data Fig. 4c,d and Supplementary Table 7). We then moved to the more complex venom of *Dendroaspis viridis*, which contains sNTx and lNTx as well as AgTx and Og XI^25^. Pre-incubation of venom with a mixture of V_H_H5 a-sNTx, V_H_H9 a-lNTx, V_H_H15 a-Og XI and V_H_H13 a-AgTx protected all mice (Extended Data Fig. 4e and Supplementary Table 7), qualifying these four V_H_Hs for inclusion in the recombinant antivenom. Next, we also wanted to ensure the neutralization of black mamba venom (*Dendroaspis polylepis*), which is rich in dendrotoxins from the KUN family but also contains sNTx and lNTx. We observed that adding V_H_H17 a-KUN to the four V_H_Hs resulted in the survival of all mice when challenged with *D. polylepis* venom (Extended Data Fig. 4f and Supplementary Table 7). To evaluate the necessity of V_H_H15 a-Og XI and V_H_H13 a-AgTx, a mixture of V_H_H5 a-sNTx, V_H_H9 a-lNTx and V_H_H17 a-KUN was tested against *D. viridis* and *Dendroaspis jamesoni* venom. Although mice challenged with *D. viridis* venom all survived, they showed signs of lethargy, and only two mice survived after being challenged with *D. jamesoni* venom, illustrating that full neutralization is not achieved without V_H_H15 a-Og XI and V_H_H13 a-AgTx V_H_Hs (Extended Data Fig. 4g and Supplementary Table 7). Finally, we evaluated the neutralization of PLA_2_ and CTx using *Naja nigricollis* venom and observed that a combination of V_H_H20 a-PLA_2_ and two anti-CTx V_H_Hs (V_H_H1 a-CTx and V_H_H4 a-CTx), which show different binding patterns to CTxs and therefore might provide broader neutralization than each of the anti-CTx V_H_Hs alone, protected all mice from lethal venom effects (Extended Data Fig. 4h and Supplementary Table 7). In total, we selected eight lead V_H_Hs targeting seven medically important toxin subfamilies and combined these into an experimental polyvalent recombinant antivenom.

**Fig. 4:**
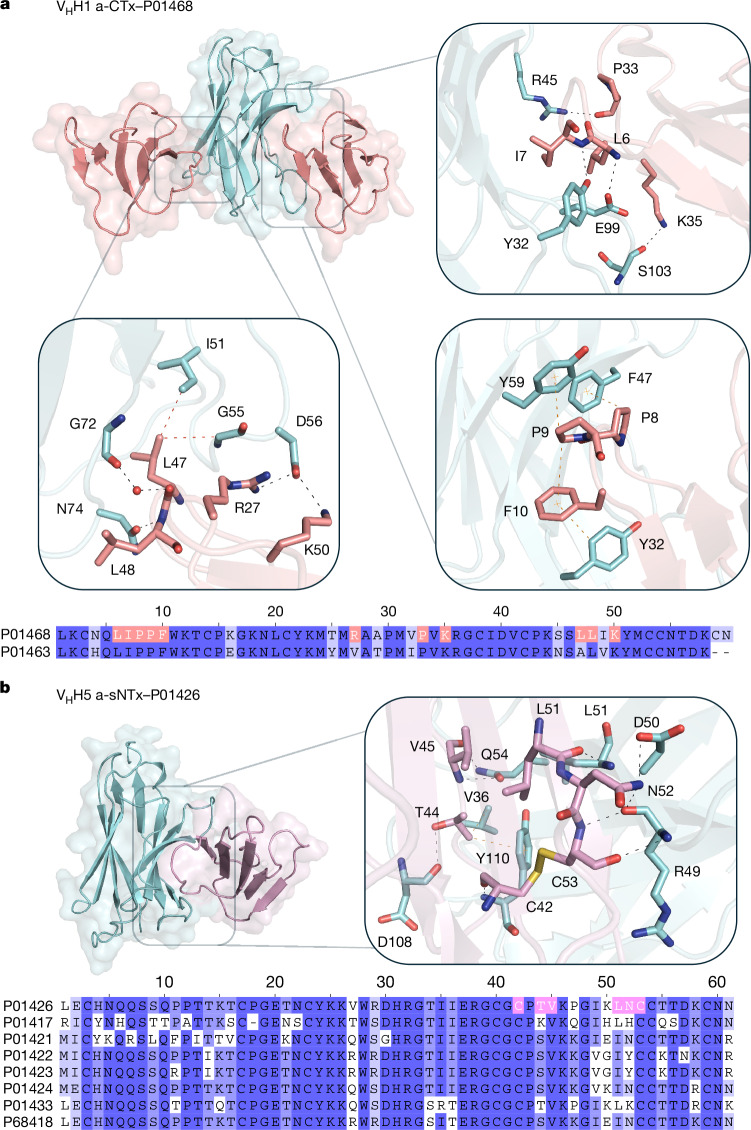
Interactions between V_H_Hs and target toxins in co-crystal structures. **a**,**b**, The biparatopic V_H_H1 a-CTx (light blue) binding two different epitopes of cardiotoxin (UniProt: P01468) (salmon) (**a**) and V_H_H5 a-sNTx (light blue) in complex with short neurotoxin 1 (P01426) (purple) (**b**). Magnified views illustrate hydrogen bonds (black dotted lines), π–π and π–CH interactions (orange dotted lines) and van der Waals interactions (red dotted lines). Sequence alignments of the verified target toxins of each V_H_H are shown under their structures. The epitope residues of the target toxin present in the co-crystal structure are highlighted in salmon and pink, respectively.

**Extended Data Fig. 5:**
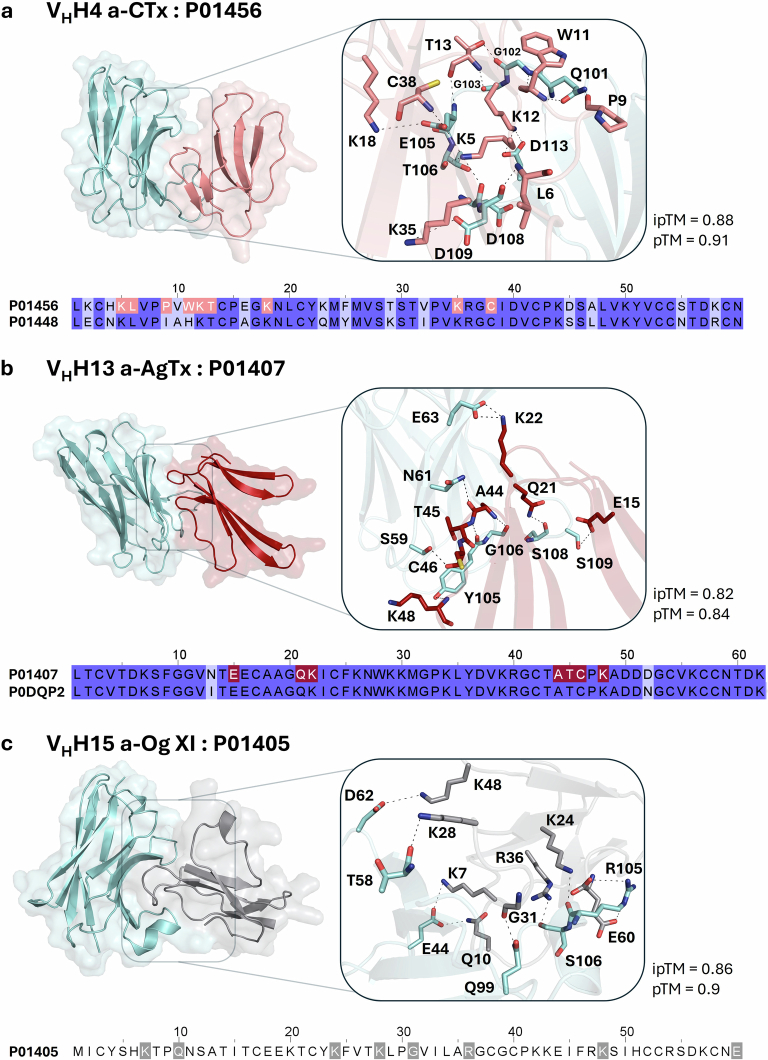
In silico predictions of V_H_H-toxin interactions. **a**, V_H_H4 a-CTx (light blue) with cytotoxin 1 (P01456) (salmon), **b**, V_H_H13 a-AgTx (light blue) with S2C4 (P01407) (dark red), and **c**, V_H_H15 a-Og XI (light blue) with Toxin 4.9.6 (P01405) (grey). The close-ups show the predicted hydrogen bonds (black dotted lines), π-π and π-C_H_ interactions (orange dotted lines), and van der Waals interactions (red dotted lines). Sequences or sequence alignments of the verified target toxins of each respective V_H_H are shown under their structures. Here, the epitope residues of the target toxins used in the in silico prediction are highlighted in corresponding colors. The respective ipTM and pTM scores of each prediction are shown next to the close-ups.

**Extended Data Fig. 6:**
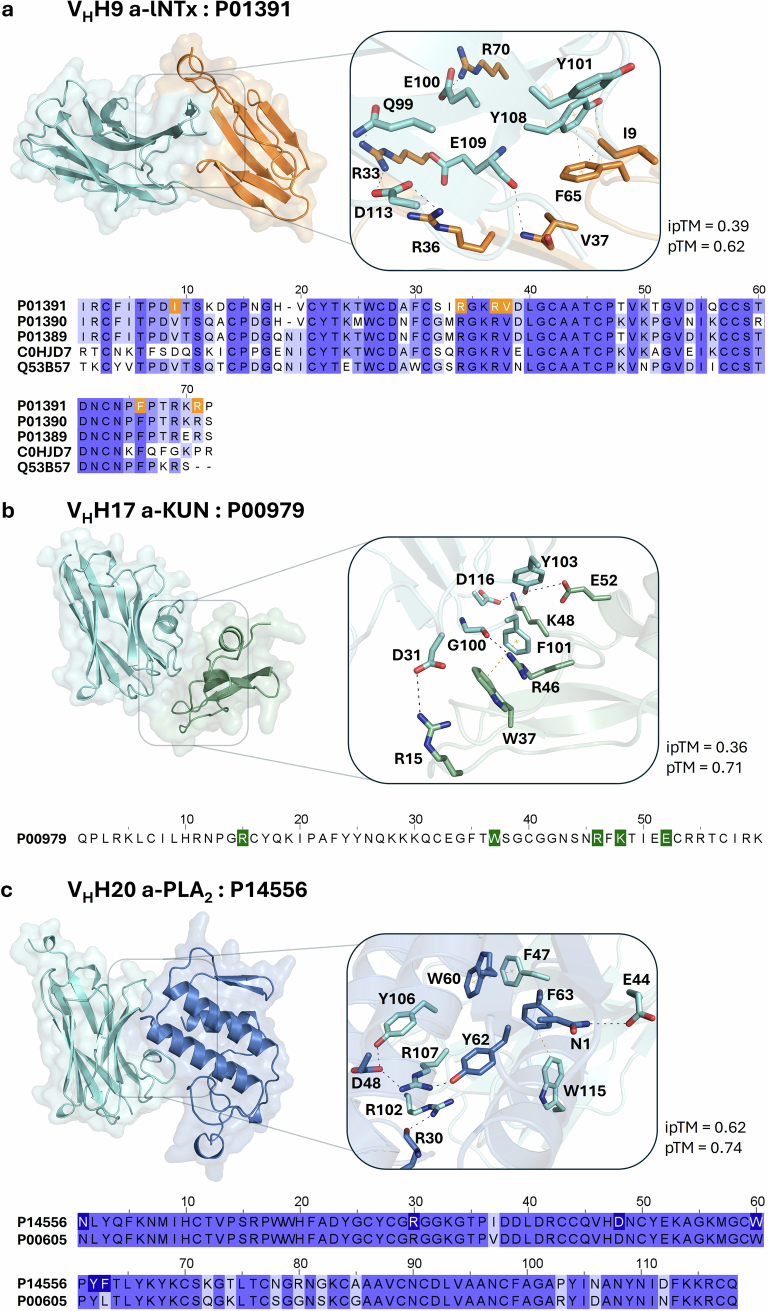
In silico predicted interactions between V_H_Hs and toxins. **a**, V_H_H9 a-lNTx (light blue) with α-cobratoxin (P01391) (orange), **b**, V_H_H17 a-KUN (light blue) with dendrotoxin I (P00979) (green), and **c**, V_H_H20 a-PLA_2_ (light blue) with basic phospholipase A_2_ nigexine (P14556) (dark blue). The close-ups show the predicted hydrogen bonds (black dotted lines), π-π and π-C_H_ interactions (orange dotted lines), and van der Waals interactions (red dotted lines). Sequences or sequence alignments of the verified target toxins of each respective V_H_H are shown below their structures. Here, the epitope residues of the target toxins used in the in silico prediction are highlighted in corresponding colors. The respective ipTM and pTM scores of each prediction are shown next to the close-ups.

**Extended Data Fig. 7:**
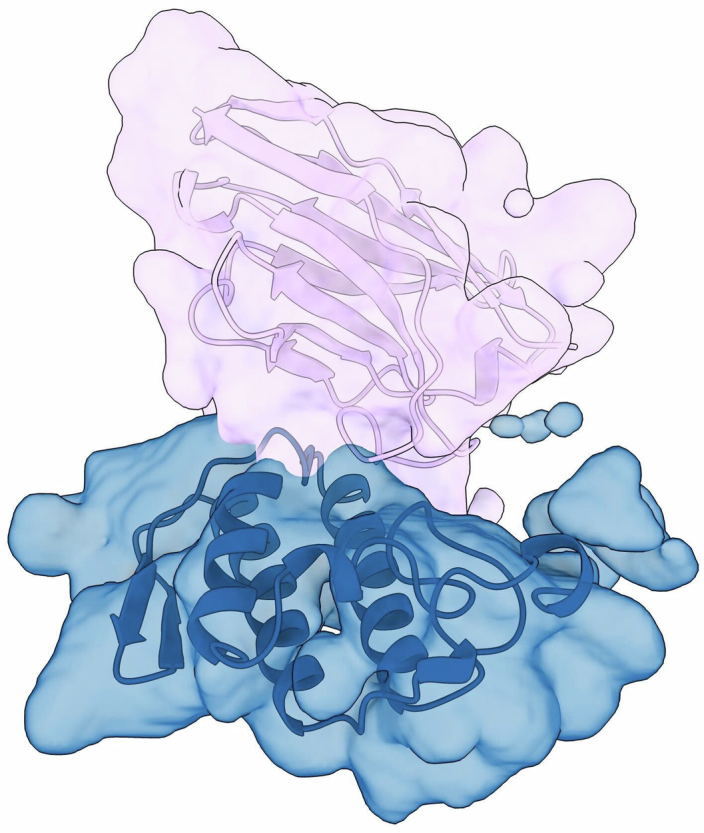
Low-resolution cryo-EM map. Indicating the binding mode of V_H_H20 a-PLA_2_ (purple) in complex with basic phospholipase A_2_ nigexine (P14556) (dark blue) with the docked AlphaFold 3 model, shown as ribbons.

To gain insights into the molecular basis of the broad neutralization of the eight lead V_H_Hs, co-crystallization, cryo-EM and in silico structural modelling predictions of the V_H_Hs in complex with one of their target toxins were used to determine their binding interactions. These experiments indicated that the V_H_Hs interact with residues that are predominantly conserved across the target toxins that they neutralize (Fig. 4 and Extended Data Figs. 5a–c, 6a–c and 7) and that the V_H_H–sNTx and V_H_H–lNTX interactions are similar to those previously reported^14,17,41^. Of note, we observed that V_H_H1 a-CTx was biparatopic, which may explain why this V_H_H was better at neutralizing cell cytotoxicity than V_H_H4 a-CTx (Fig. 3b). To our knowledge, such biparatopic behaviour has not previously been reported and is worthy of further investigation.

## The V_H_H cocktail prevents lethality

**Fig. 5:**
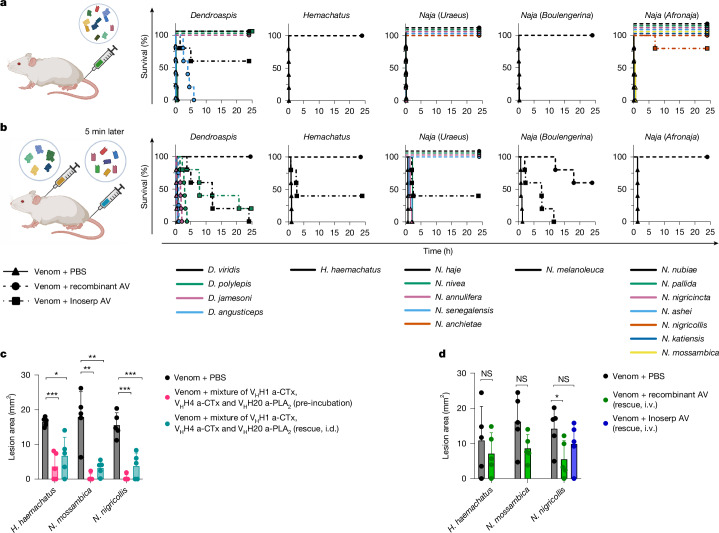
In vivo neutralization of venom-induced lethality and dermonecrosis. **a**, Neutralization efficacy of the recombinant antivenom (AV) against venom-induced lethality caused by 18 elapid venoms in a pre-incubation setup. Venom and the recombinant antivenom were pre-incubated before intravenous injection. **b**, Neutralization efficacy of the recombinant antivenom against venom-induced lethality caused by 11 snake venoms in a rescue setup. Venom was injected subcutaneously followed by intravenous injection of the recombinant antivenom 5 min later. In both setups, Inoserp PAN-AFRICA antivenom was included for comparison. **c**, Neutralization of venom-induced dermonecrosis with a mixture of three V_H_Hs (V_H_H1 a-CTx, V_H_H4 a-CTx and V_H_H20 a-PLA_2_) against three representative cytotoxic venoms from *H. haemachatus*, *N. mossambica* and *N. nigricollis*, in both pre-incubation and rescue setups. In the pre-incubation setup, the V_H_Hs and venom were pre-incubated before intradermal (i.d.) injection, and in the rescue setup, the V_H_H mixture was injected into the same region as the venom injection 15 min after venom injection. The lesion size was measured 72 h post-injection and results are shown as mean ± s.d. **d**, The neutralization efficacy of the recombinant antivenom against dermonecrosis caused by the same three venoms in a second rescue setup. In this rescue setup, venom was injected intradermally and the recombinant antivenom was injected intravenously (i.v.) 15 min later. The lesion size was measured 48 h post-injection and results are shown as mean ± s.d. For *N. nigricollis* venom, in addition to the recombinant antivenom, Inoserp PAN-AFRICA antivenom was included for comparison. **c**,**d**, Lesion sizes were compared to PBS controls using Welch’s *t*-test. Normality (Shapiro–Wilk) and outliers (ROUT) were assessed. Significance was set at *α* = 0.05. *P ≤ 0.05, **P < 0.01, ***P < 0.001; NS, not significant. *n* = 5 mice. Created in BioRender. Burlet, N. (2025) https://BioRender.com/rkd7bzp.

To assess whether the recombinant antivenom (that is, the defined cocktail of 8 selected V_H_Hs) could neutralize venom-induced lethality caused by all of the 18 most medically relevant snakes in sub-Saharan Africa, we performed a series of pre-incubation experiments in mice^39^. In this set of experiments, we first determined the LD_50_s for the whole venoms injected intravenously (Supplementary Table 6). Then, three times the LD_50_s of the venoms were pre-incubated with the same dose of recombinant antivenom and administered intravenously to groups of five mice. The composition and dose of the recombinant antivenom were kept consistent across all venoms to simulate a real product being deployed in Africa, with the dose corresponding to at least a 1:10 molar ratio between each targeted toxin (sub)family and the respective V_H_H (Supplementary Table 8). Survival and signs of envenoming were monitored over a 24 h period post-injection. We observed that the recombinant antivenom prevented venom-induced lethality for all included species except *D. angusticeps* (Fig. 5a). No evident signs of envenoming were observed in mice that were administered recombinant antivenom pre-incubated with venoms from *D. jamesoni*, *H. haemachatus*, *Naja ashei*, *Naja katiensis*,* Naja mossambica*, *Naja nigricincta*,* N. nigricollis*,* Naja nubiae*, *Naja pallida*,* Naja anchietae*,* N. haje*, *Naja nivea* and *Naja senegalensis*. In mice injected with the recombinant antivenom pre-incubated with the venoms from *N. melanoleuca* and *D. viridis*, only minor signs of envenoming, including closed eyes and periods of lethargy interspersed with episodes of excessive grooming, were observed. For mice injected with recombinant antivenom pre-incubated with *Naja annulifera* venom, similar signs of envenoming appeared after 15 h. The venom from *D. polylepis* pre-incubated with the recombinant antivenom caused an initial state of severe lethargy, which disappeared after 1–2 min. We speculate that this rapid transient effect could be caused by small molecules in the venom such as adenosine or acetylcholine, which the recombinant antivenom is not designed to neutralize^42^. For *D. angusticeps* venom, we observed that the recombinant antivenom prolonged the survival of mice to between 3 and 6 h. This venom is rich in muscarinic toxins, fasciculins and synergistically acting toxins^43^, and we speculate that the synergy between these toxins, which are not targeted by the recombinant antivenom, could be responsible for the observed delayed lethality.

To better evaluate the efficacy of the recombinant antivenom in a scenario that mimics real snakebite envenoming, we further performed rescue experiments using 11 venoms from 4 different (sub)genera^12,44^. To perform these experiments, we first determined the subcutaneous venom LD_50_s. However, when attempting to determine subcutaneous LD_50_s for *Afronaja* venoms, we observed severe muscle and skin damage at the injection sites, and mice had to be euthanized owing to ethical considerations. Given that local tissue damage is the most clinically relevant effect of *Afronaja* envenomings, we deemed rescue-from-lethality experiments for these species to be ethically unjustifiable. For the remaining species, we administered three times the LD_50_ of the venom subcutaneously (Supplementary Table 6), followed by intravenous administration of the recombinant antivenom 5 min later, and monitored survival and signs of envenoming over a 24 h period post-injection (Supplementary Table 9). In these rescue experiments, the dose of the recombinant antivenom was increased compared with the pre-incubation experiments relative to the increase in LD_50_s that was observed when switching from intravenous to subcutaneous administration of venom (Supplementary Table 9). For comparison, the commercial Inoserp PAN-AFRICA antivenom was included at a dose recommended by the manufacturer to neutralize three times the LD_50_ of the venom. We observed that the recombinant antivenom completely prevented lethality induced by the venoms from *N. haje*,* N. annulifera*,* N. nivea*,* N. senegalensis*,* N. nubiae* and *H. haemachatus* (Fig. 5b and Supplementary Table 9), and the mice showed no signs of envenoming. Furthermore, the recombinant antivenom also prevented lethality induced by the venom of *D. viridis* (Fig. 5b and Supplementary Table 9), although we observed signs of envenoming, including limited movement, approximately 4 h after venom injection and continuing throughout the experiment. In addition, after 20 h, four out of five mice developed swelling and haemorrhage of the eyeballs, and one mouse developed an increased abdominal volume. For *N. melanoleuca* venom, partial neutralization was observed, with three out of five mice surviving, and the time of death being substantially extended for the other two mice (Fig. 5b and Supplementary Table 9). The surviving mice presented similar signs of envenoming as observed for *D. viridis*, but did not show an increased abdominal volume. Finally, for *D. polylepis* venom, the recombinant antivenom delayed the time of death from approximately 0.5 h in the venom-only control mice to 2 h; for *D. jamesoni* venom, the time of death was delayed from approximately 0.5 h to 1 h; and no protective effect was seen with the venom from *D. angusticeps* (Fig. 5b and Supplementary Table 9). On the basis of the difference between pre-incubation and rescue experiments for *D. polylepis* and *D. jamesoni*, we hypothesize that this is owing to the complex interplay between venom and antivenom pharmacokinetics. It has been observed that the ability of antivenoms to redistribute and neutralize the toxins once they have reached their target strongly depends on the affinity of the antibodies present in the antivenom for the toxin as well as the affinity of the toxin for its respective target within the tissue^45^. Furthermore, a delayed release of the toxins from the subcutaneous injection site, due to the depot effect, could occur after the V_H_Hs have been cleared from the circulation, which could have resulted in a recurrence of envenoming signs in the absence of repeated therapeutic dosing^46,47^. Notably, in the rescue setting, the Inoserp PAN-AFRICA antivenom showed only partial neutralization of all of the tested venoms and an extension of time of death for the venom from *N. melanoleuca*, demonstrating that, except for *D. polylepis*, the recombinant antivenom performed better than the commercial antivenom on all included venoms at the tested doses (Fig. 5b and Supplementary Table 9).

## The V_H_H cocktail prevents dermonecrosis

**Extended Data Fig. 8:**
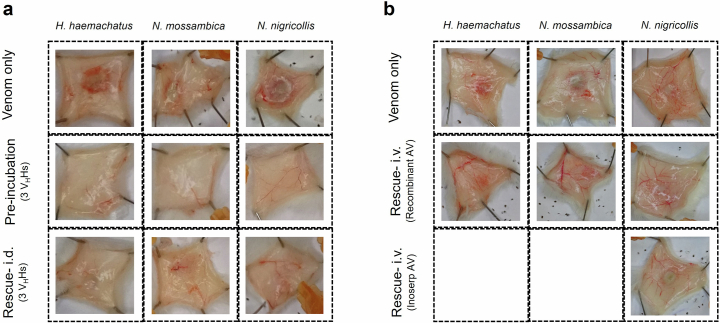
Evaluation of venom-induced dermonecrosis and the inhibitory effect of V_H_Hs antivenom. **a**, Macroscopic view of representative inner skin samples from the experimental setup with the 72-h post-injection endpoint. Venom (MND) was injected alone, pre-incubated with a mixture of 3 V_H_Hs (V_H_H1 a-CTx, V_H_H4 a-CTx, and V_H_H20 a-PLA_2_) or venom injected followed 15 min later by an intradermal injection of the same mixture of 3 V_H_Hs. **b**, Macroscopic view of representative inner skin samples from the experimental setup with the 48-h post-injection endpoint. Venom (MND) was injected, followed 15 min later by an intravenous (i.v.) injection of PBS, recombinant antivenom, or Inoserp PAN-AFRICA antivenom.

Current plasma-derived antivenoms are typically poor at preventing local tissue damage, resulting in a high morbidity rate, including limbs lost, in snakebite victims^8–10^. In elapid snakes in sub-Saharan Africa, local tissue damage is primarily associated with spitting snake species (*Afronaja* and *Hemachatus* spp.), and CTx and PLA_2_ have the most important role^38^. We therefore examined the ability of our two anti-CTx V_H_Hs (V_H_H1 a-CTx and V_H_H4 a-CTx) and anti-PLA_2_ V_H_H (V_H_H20 a-PLA_2_) to prevent venom-induced dermonecrosis caused by *N. mossambica*, *N. nigricollis* and *H. haemachatus* using an in vivo pre-incubation setup. We mixed the three V_H_Hs and pre-incubated this mixture with each of the venoms before intradermal injection and measured the lesion size after 72 h. The V_H_H mixture significantly reduced the dermonecrotic lesion areas, and all but one mouse per treatment group showed a complete absence of lesion for the two *Naja* venoms (Fig. 5c). We then moved to a rescue setup, where the V_H_H mixture was delivered intradermally to the same injection region 15 min after the venom. Despite observations of rapid discoloration at the injection site within the 15 min treatment window, the V_H_H mixture significantly reduced the size of the dermonecrotic lesions caused by each of the three venoms (Fig. 5c and Extended Data Fig. 8a).

After demonstrating the efficacy of the mixture of V_H_H1 a-CTx, V_H_H4 a-CTx and V_H_H20 a-PLA_2_ in both pre-incubation and rescue setups, we evaluated the recombinant antivenom (containing the eight V_H_Hs) in another rescue assay, where the recombinant antivenom was delivered intravenously 15 min after intradermal injection of the venom. Inoserp PAN-AFRICA antivenom was included for comparison with *N. nigricollis* venom at a dose recommended by the manufacturer to neutralize three times the LD_50_ of the venom. The recombinant antivenom reduced the size of the lesions caused by each of the three venoms at the 48 h experimental endpoint, although these results were statistically significant only for *N. nigricollis* (Fig. 5d and Extended Data Fig. 8b). In comparison, we observed less, and statistically insignificant, reduction of lesion size for Inoserp PAN-AFRICA antivenom on the tested venom from *N. nigricollis* (Fig. 5d), demonstrating that the recombinant antivenom performs better than the existing treatment in this model at the tested doses.

## Discussion

Previous studies have shown that broadly neutralizing antibodies or V_H_Hs against snake toxins can be identified using phage or yeast display technology^12,14,18,20,34,41,48,49^. However, although these studies demonstrated that rodents challenged with venoms dominated by neurotoxins can be rescued using such antibodies^14,18^ and V_H_Hs^20^, they represent only an initial proof of principle when viewed from a product development or clinical perspective. Notably, all neutralized venoms have been dominated by only one or two toxin families, and all but three studies^17,18,20^ have reported the use of only a single monoclonal antibody or V_H_H to neutralize a whole venom. In this study, we extend earlier work and demonstrate how an experimental polyvalent recombinant antivenom can be developed using as few as eight V_H_Hs targeting toxins from seven different protein subfamilies to achieve continent-wide elapid species coverage. We demonstrate its in vivo efficacy on 18 elapid venoms using the WHO-recommended pre-incubation model and further confirm the efficacy on 11 of the venoms in a more challenging rescue model, which more closely mimics a real snakebite scenario^44^. Furthermore, at the tested doses, this experimental recombinant antivenom performs better than the commercial plasma-derived antivenom, Inoserp PAN-AFRICA, in preventing both dermonecrosis and lethality across all snake species tested, apart from *D. polylepis*. By showing that an efficacious polyvalent recombinant antivenom with unprecedented broad species coverage can be constructed using only eight V_H_Hs, we challenge the common belief that only polyclonal antibodies can overcome and neutralize the complexity of snake venoms. Thereby, we answer the long-standing and important question in toxinology of how few antibodies are sufficient to develop a recombinant antivenom with clinical utility. Our work thus solidifies our previous working hypotheses that it is indeed possible to unravel the challenging complexity of venoms through proteomic analysis^29^ and identify which toxins are sufficient to neutralize in order to develop effective antivenom products^28,50,51^.

In addition to mortality, we demonstrate the efficacy of the experimental recombinant antivenom in reducing dermonecrosis caused by the venoms of the spitting elapid snakes, *N. nigricollis*, *N. mossambica* and *H. haemachatus*, in a pre-incubation model and in two rescue models. Although plasma-derived antivenoms have demonstrated efficacy in pre-incubation models of dermonecrosis, they have rarely been assessed in rescue models^52^. Further, it is well established that antivenoms are largely ineffective in preventing severe local tissue damage unless given rapidly after a bite^52,53^, which is typically attributed to the limited ability of whole IgG molecules or IgG fragments administered intravenously to penetrate deep tissue and the speed with which local tissue damage develops. In comparison, we here show that delayed local administration (intradermal) of toxin-specific V_H_Hs significantly reduces dermonecrosis caused by different elapid snake venoms, analogous to recent findings with the PLA_2_-inhibiting small molecule varespladib and the CTx-inhibiting heparinoid tinzaparin^54,55^. In addition, the recombinant antivenom further provides significant protection against dermonecrosis caused by *N. nigricollis* venom in a rescue model when administered intravenously. This finding is particularly promising given the rapid onset of toxicity caused by locally acting toxins that induce tissue damage, the ineffectiveness of current antivenoms (Fig. 5d) and the lack of protection previously offered by intravenous varespladib^54^. Of note, neither V_H_H20 a-PLA_2_, V_H_H1 a-CTx nor V_H_H4 a-CTx neutralizes the *H. haemachatus* venom in vitro in the cell viability or phospholipase activity assays. These findings highlight the need for further investigation to elucidate the mechanisms underlying the observed neutralization of local tissue damage in mouse experiments.

Regarding design features for our experimental recombinant antivenom, we chose to use the V_H_H format, as this simple antibody format could provide a recombinant antivenom with several advantages. First of all, the use of this clinically proven antibody format might aid in the development of a safe product with a low risk of causing severe immunological reactions upon administration owing to the typically low immunogenicity observed for V_H_Hs^5^. An enhanced safety profile may enable earlier treatment of patients by removing the need to wait for clinical syndromes to manifest before administration of antivenom. Second, V_H_Hs are known to have a remarkably high biophysical stability, which is likely to translate into a long shelf-life that might be advantageous for products that may need to be stockpiled for longer periods, possibly in humid or high-temperature conditions. Third, the small size of V_H_Hs enables them to rapidly distribute from the bloodstream into the surrounding tissues and thereby neutralize toxins at the bite site or site of action. Finally, the small size of V_H_Hs compared to full-length IgGs provides them with a large neutralization capacity per mass of antivenom^56^. Combined with the possibility of manufacturing V_H_Hs using low-cost microbial expression systems, this might facilitate a lower cost of goods for treatment. In turn, a lower cost of goods could positively affect affordability and make antivenoms more accessible to people in low- and middle-income countries who disproportionally suffer the greatest burden of snakebite^57^.

Despite these advantages, recombinant V_H_Hs are also associated with certain limitations. Most importantly, the short half-life of V_H_Hs in circulation could potentially limit their sustained action over time, which could prove to be problematic, as snake toxins may exit the bite site and enter circulation long after the snakebite incident owing to the depot effect^46,47^. However, it is also possible that the ability of V_H_Hs to rapidly penetrate into deep tissue could counter this effect, as it might allow the V_H_Hs to neutralize the toxins before they are released into circulation. To study this complex interplay between pharmacodynamics of the V_H_Hs and the toxicokinetics of the venom components, large animal experiments are probably required. If it is found necessary, repeated pharmacokinetic-informed dosing can be applied or the half-life of the V_H_Hs could possibly be extended through various antibody engineering techniques, such as multimerization or linking them to a human IgG Fc domain^20^. Finally, it is possible that our cocktail could be further simplified by reducing the number of molecules included, which is likely to be beneficial from a manufacturing perspective. This could be achieved by improving the broadly neutralizing capacity of some of our V_H_Hs, such as those targeting CTx, and including only one broadly neutralizing V_H_H per toxin (sub)family. Moreover, engineering heterodivalent or trivalent V_H_H constructs^58,59^, through fusion of two or three V_H_Hs, could reduce the number of individual molecules required to neutralize the described toxin specificities, which could further simplify manufacturing. However, the total amount of material needed in terms of mass would remain the same (or become higher), and we speculate that the favourable deep tissue-penetrating properties of the small monomeric V_H_Hs risk being compromised, based on what is known from the oncology field^60^. Nevertheless, further refinement of the cocktail is likely to be possible and should be further investigated alongside the development of a manufacturing strategy and an assessment of whether the V_H_Hs might be useful for neutralizing venoms from related Asian elapids.

Although our work here focused on developing a defined oligoclonal mixture of V_H_Hs against African elapid venoms, the strategy utilized for antibody discovery and therapeutic prototype design holds promise for broader applications. Specifically, it may be valuable in clinical contexts where targeting multiple isoforms across both closely and distantly related protein families is essential. We speculate that, beyond other animal envenomings, the approaches described here could be useful for the development of advanced therapies against infectious diseases or complex immunological or endocrinological disorders.

## Methods

### Construction of an immune V_H_H-displaying phage library

Immune V_H_H-displaying phage libraries were constructed at the VIB nanobody core (Brussels, Belgium) as described^20^. To generate V_H_H-displaying phage libraries, 1 alpaca and 1 llama were injected subcutaneously at bi-weekly intervals across 8 time points with increasing doses of venom mixtures from the 18 most medically relevant elapid snakes in sub-Saharan Africa: *D. angusticeps*, *D. jamesoni*, *D. polylepis*, *D. viridis*, *N. anchietae*, *N. annulifera*, *N. ashei*, *N. haje*, *N. katiensis*, *N. melanoleuca*, *N. mossambica*, *N. nigricincta*, *N. nigricollis*, *N. nivea*, *N. nubiae*, *N. pallida*, *N. senegalensis* and *H. haemachatus*. Following the initial series of injections, 3 additional booster injections were administered at 52, 54 and 60 weeks after the first immunization (Supplementary Table 2 summarizes the detailed immunization schedule). For library generation, blood samples were collected on days 5 and 8 following the first set of 4 injections. The two blood samples from each animal were pooled separately and individual libraries were prepared for each animal. A total of 6 V_H_H-displaying phage libraries (1 library per time point and animal) was prepared by pooling the total RNA samples after days 46 and 49 (library A), 102 and 105 (library B) and days 5 and 8 following the final booster injections (library C).

### Purification and biotinylation of the venom fractions and toxins

Cardiotoxin (P01468) from *N. pallida*, α-cobratoxin (P01391) from *N. kaouthia*, α-short-chain neurotoxin (P01426) from *N. pallida*, and whole venoms from the above-mentioned 18 elapid snakes were purchased in lyophilized form from Latoxan (catalogue numbers and origin of the specimens can be found in Supplementary Table 1). Venom fractions containing short-chain neurotoxins (sNTx), long-chain neurotoxins (lNTx), cytotoxins (CTx), Og XI, AgTx, PLA_2_ and dendrotoxins (DTx) were isolated from the whole venoms using RP-HPLC (Agilent 1200) with a C_18_ column (250 × 4.6 mm, 5 μm particle; Teknokroma). 1 mg of venom solubilized in 100 μl solution A (MilliQ water supplemented with 0.1% trifluoroacetic acid (TFA)) was applied to the column and elution was performed at a rate of 1 ml min ^− 1^ using solution A and a gradient towards solution B (acetonitrile supplemented with 0.1% TFA): 0% B for 15 min, 0–15% B over 15 min, 15–45% B over 60 min, 45–70% B over 10 min, and 70% B over 9 min, as described^69^. Fractions were collected and the solvent evaporated using a vacuum centrifuge. The venom fractions purified via RP-HPLC and toxins bought from Latoxan were dissolved in phosphate buffered saline (PBS: 137 mM NaCl, 3 mM KCl, 8 mM Na_2_HPO_4_**·**2H_2_O, 1.4 mM KH_2_PO_4_, pH 7.4) and biotinylated by amine coupling using a 1:1 to 1:3 molar ratio of venom fraction or toxin to EZ-Link NHS-PEG_4_-Biotin reagent (Thermo Scientific, A39259), as described^34^. Free biotin was removed using 2 or 4 kDa MWCO ultracentrifugation membranes (Vivacon 500, VN01H91 and Amicon Ultra-4, UFC8000324, respectively) in accordance with the manufacturers’ guidelines. Following purification, the degree of biotinylation was analysed by matrix-assisted laser desorption/ionization time-of-flight (MALDI-TOF) mass spectrometry using ProteoMass Protein MALDI-MS Calibration Kit (Sigma-Aldrich, MSCAL3) and an Ultraflex II TOF/TOF spectrometer (Bruker Daltonics), as described^70^.

### Proteomics analysis of the selected venom fractions

From each venom fraction, 5 µg was diluted in 50 mM ammonium bicarbonate to a total volume of 25 µl. The samples were reduced and alkylated by 10 mM TCEP and 40 mM chloroacetamide before digestion with either GluC or trypsin in an enzyme-to-protein ratio of 1:100. Samples were incubated overnight at 37 °C, after which the digestion was stopped by addition of 2% TFA for a final concentration of 1%. The samples were desalted with SOLAµ SPE plate (HRP, Thermo) C18 columns, following the same procedure as described^71^. Dried peptides were reconstituted in 12 µl 2% acetonitrile, 1% TFA, and an estimated 500 ng of peptides was used for mass spectrometry analysis.

Peptides were loaded onto a 2 cm C18 trap column (ThermoFisher 164946), connected in-line to a 15 cm C18 reverse-phase analytical column (Thermo EasySpray ES904) using 100% solvent A (0.1% formic acid in water) at 750 bar, using the Thermo EasyLC 1200 HPLC system, and the column oven operating at 35 °C. Peptides were eluted over a 35 min gradient ranging from 6 to 60% of solvent B (80% acetonitrile, 0.1% formic acid) at 250 nl min^−1^, and the Q-Exactive instrument (Thermo Fisher Scientific) was run in a DD-MS2 top10 method. Full mass spectra were collected at a resolution of 70,000, with an AGC target of 3 × 10^6^ or maximum injection time of 20 ms and a scan range of 300–1,750 *m*/*z*. The MS2 spectra were obtained at a resolution of 17,500, with an AGC target value of 1 × 10^6^ or maximum injection time of 60 ms, a normalized collision energy of 25 and an intensity threshold of 1.7 × 10^4^. Dynamic exclusion was set to 60 s, and ions with a charge state <2 or unknown were excluded.

The raw data from all fractions were analysed with Proteome Discoverer v.2.4. The data were searched against all snake venom proteins (retrieved from Uniprot, 2,263 sequences, accessed 9 November 2021). The trypsin-digested fractions were searched with tryptic specificity, while the GluC-digested fractions were searched with GluC specificity, with two maximum missed cleavages allowed for both proteases. Minimum and maximum peptide lengths were set to 7 and 40, respectively. Precursor mass tolerance was 10 ppm, and fragment mass tolerance was 0.02 Da. Methionine oxidation (+15.995 Da) was set as dynamic modification, while initiator methionine loss (−131.040 Da), acetylation (+42.011 Da), or the combination of methionine loss and acetylation (−89.030 Da) were included as dynamic modifications for the protein terminus. Cysteine carbamidomethylation (+57.021 Da) was added as a static modification. Peptide-spectrum matching was performed with Sequest HT, and false discovery rate (FDR) control with Percolator (0.01 strict and 0.05 relaxed target FDR). FDR was also controlled at the peptide and protein levels with the same target FDRs. Proteins were quantified based on the unique and razor peptides, using the Minora Feature Detector and the Precursor Ions Quantifier nodes with default settings, normalizing abundance to the total peptide amount in each mass spectrometry run and scaling abundance values on the average of all runs.

### Clustering toxins on the basis of sequence identity

A sequence similarity network (SSN) was made with the Enzyme Function Initiative-Enzyme Similarity Tool (EFI-EST)^72,73^. A fasta file containing the UniProt sequences of each discovered toxin from the whole venom of the included 18 elapid snakes was used by the tool to perform an all-by-all BLAST to obtain similarities between sequence pairs. Clustering of toxins with a minimum sequence identity of 70% was subsequently performed by using an alignment score threshold during SSN Finalization that corresponds to 70% identity in the ‘percent identity vs alignment score box plot’ in the Dataset Analysis tab. The obtained SSN was visualized with Cytoscape.

### Solution-based phage display selections

V_H_H-displaying phage libraries were incubated with biotinylated venom fractions or toxins for 2 h at ambient temperature, with end-over-end rotation (Supplementary Table 3 summarizes the final concentration of target toxins and the libraries used in each selection round). Streptavidin coated Dynabeads (M-280, Fisher Scientific, 10465723) were blocked in PBS containing 3% non-fat dried milk powder for 1 h with end-over-end rotation, before addition to the target toxins mixed with the phage library. In each selection round, a background control was included where no antigen was mixed with the phage library. Subsequently, a KingFisher Flex system (Thermo Scientific, 711-82573) was used to wash the beads 3 times with PBST (PBS + 0.1% Tween) and 3 times with PBS, before eluting the bound phages in 120 µl of 0.1 mg ml^−1^ trypsin (Sigma-Aldrich, T9201-500MG) in phage elution buffer (50 mM Tris, 1 mM CaCl_2_, pH 8.0). The eluted phages were amplified using the M13KO7 helper phage and concentrated by polyethylene glycol precipitation.

### Subcloning, screening, and sequencing of V_H_Hs

Phagemids from the chosen selection outputs (Supplementary Table 3) were purified using the GeneJET Plasmid MiniPrep Kit (Thermo Fisher, K0503) according to the manufacturer’s protocol. The V_H_H-encoding genes were subcloned into the pBDS100 expression vector using the PstI and Eco91I restriction enzymes (New England Biolabs). Following transformation into the *E. coli* strain BL21 (DE3) (New England Biolabs), at least 184 individual colonies were picked from each chosen selection output and used for the expression of soluble V_H_Hs. Auto-induction medium^74^ was used to induce V_H_H expression for 16 h at 30 °C. Thereafter, periplasmic cell extracts, containing soluble expressed V_H_Hs, were used for primary screenings in a previously described expression-normalized DELFIA^20^ using 25 nM of target toxin. Clones with a signal intensity ten times higher than the background (no addition of biotinylated target), were cherry-picked and went through a second round of screening in the expression-normalized DELFIA, against multiple target toxins. For the cross-reactive clones, a dose–response experiment was performed, where the Flag-tagged V_H_Hs in the periplasmic extracts were captured onto the wells coated with 2.5 µg ml^−1^ Flag antibody clone M2 (F3165, Sigma-Aldrich); however, instead of a single concentration, a serial dilution of target toxins (1:1,000 nM) was added. Clones displaying a signal intensity 50 times over the negative control, and/or a low EC_50_ in the dose–response curves, were Sanger sequenced (Eurofins Genomics sequencing service) using the M13Rev primer (CAGGAAACAGCTATGAC). The V_H_H frameworks and the complementarity determining regions (CDRs) were annotated using CLC Main Workbench (Qiagen) and the V_H_Hs with unique CDR sequences were produced for in vitro and in vivo assays.

### Production of V_H_Hs for in vitro and in vivo experiments

For expression of V_H_Hs at scales up to 100 ml, the periplasmic extracts containing V_H_Hs were produced as described in the screening section and then purified using Ni-resin (Sigma-Aldrich, P6611) via gravity flow. For larger-scale expressions (>250 ml), BL21 (DE3) cells, containing the plasmid encoding a unique V_H_H, were cultivated as described^20^. Thereafter, the V_H_H-containing supernatants were purified using immobilized metal ion affinity chromatography with a 2 ml column volume of Ni-NTA resin (HIS-select Nickel Affinity Gel, Sigma-Aldrich, P6611) equilibrated with PBS supplemented with 200 mM NaCl and 20 mM imidazole, pH 8.0. Elution was performed with PBS containing 200 mM NaCl and 135 mM imidazole, pH 8.0, followed by an overnight dialysis in SnakeSkin Dialysis Tubings (10 kDa MWCO, ThermoFisher Scientific, 68100) against PBS. Subsequently, V_H_Hs were concentrated using Amicon Ultra-15 centrifugal filters (3 kDa MWCO, Fisher Scientific, 10781543).

### Kinetic analysis of V_H_Hs using BLI

The binding of V_H_Hs to the venom fractions and toxins was analysed using BLI (Octet-BLI; Octet RED 96, ForteBio). Biotinylated venom fractions and toxins at a concentration of 0.5 µg ml^−1^ were captured to a target spectral shift of 0.8 nm on a streptavidin-coated BLI biosensor (Sartorius, 18-5020). A biosensor without antigen was included as a reference. V_H_Hs were prepared in running buffer (10 mM HEPES, 150 mM NaCl, 3 mM EDTA, 50 mM MES hydrate, and 0.05% P_20_ (MES-HEPES), pH 7.2). The toxin-loaded biosensors were dipped into four different V_H_H concentrations (7.5, 30, 120, 480 nM) and a control without any V_H_H. V_H_H association was measured for 600 sec, followed by measuring V_H_H dissociation in running buffer for 600 sec. Biosensors were regenerated by dipping into the regeneration buffer (10 mM Glycine, 4 M sodium chloride, pH 2.0) between each round, 5 times, for 10 sec each. For analysis, the reference BLI biosensor background was subtracted, a global model assuming a 1:1 interaction was used for fitting of the data, and calculations of kinetic parameters were all made in Octet Analysis Studio 12.2.2.26 (ForteBio).

### Patch clamp electrophysiology

Automated planar whole-cell patch clamp experiments were performed as described^20^. All experiments were performed on a Qube 384 automated patch clamp platform (Sophion Bioscience) with 384-channel, 10X mode patch chips (10 patch holes/site, site resistance 0.2 ± 0.04 MΩ). We used a human rhabdomyosarcoma cell line (American Type Culture Collection, ATCC) endogenously expressing muscle type nAChRs ((α_1_)_2_β_1_γδ) and 70 µM acetylcholine for receptor activation. We first determined the IC_80_ for the included toxins or venom fractions (sNTX-1, sNTx-3, sNTx-6, lNTx-3, lNTx-5 and lNTx-7) and used this concentration to evaluate the neutralization effect of the corresponding V_H_Hs. The V_H_Hs were used at molar ratios of 9:1 to 1:27 between toxin and V_H_H. Finally, the inhibitory effect of the toxins on the elicited acetylcholine current was normalized to the full acetylcholine response and averaged in each group (*n* = 8). The data were analysed with Sophion Analyzer v.6.6.70 (Sophion Bioscience) and GraphPad Prism 10 software.

### In vitro neutralization of cell cytotoxicity

A cell viability assay was performed as described^34^. In brief, N/TERT keratinocytes were seeded at 4,000 cells per well in 100 µl cell culture medium and incubated overnight under standard conditions. After determining the IC_50_ of each venom, the cells were subjected to a venom concentration of 2 × IC_50_, either in the absence or presence of a 1:5 molar ratio of CTx or PLA_2_ to V_H_H based on the CTx or PLA_2_ contents of each venom^29^, followed by a 24 h incubation step. Thereafter, the CellTiter-Glo luminescent cell viability assay (Promega) was performed in triplicate according to the manufacturer’s protocol. A maximal cell death control was included, where the cell culture medium was supplemented with 0.01% Tween 20 to disrupt the cells. In addition, a maximum cell viability control was included, with cell culture medium supplemented with PBS, as well as a V_H_H control, where cells were incubated with the highest tested V_H_H concentration without venom, to confirm that the V_H_Hs alone do not affect cell viability. The data were visualized with GraphPad Prism 10 software.

### In vitro neutralization of PLA_2_ enzymatic activity

Venom concentration inducing half of the maximum PLA_2_ enzymatic activity (EC_50_) was determined as described^29^. For inhibitory dose–response curves, V_H_Hs were diluted to 16 µM, followed by a twofold serial dilution in 10 steps. 50 µl of snake venom at a concentration of 4 × EC_50_ was mixed with the serial dilutions of the V_H_Hs and then incubated at room temperature for 30 min. The enzymatic reaction was started by adding 100 µl of 0.5 mM NOBA into the mixture. Final concentrations of the individual components in the enzymatic activity assays were 0.25 mM NOBA, and a twofold serial dilution of the V_H_Hs with the highest concentration set at 4 µM. After adding NOBA to the wells, plates were shaken at 300 rpm for 2 min, and then incubated at 37 °C for 40 min. Finally, the plates were centrifuged at 4,000*g* at 4 °C for 3 min, and absorbance was measured at 25 °C at 405 nm using a Multimode Microplate Reader (VICTOR Nivo, HH35000500). The experiments were performed in duplicate and the absorbance averages were determined after subtracting a blank control containing no venom. The data were analysed using the Victor Nivo Control software v.5.1.0 and Graphpad Prism 10 software with a nonlinear fit using ‘Sigmoidal, 4PL, X is concentration’.

### Co-crystallization of V_H_Hs and toxins

Lyophilized toxins and vacuum-dried venom fractions were reconstituted at 10 mg ml^−1^ in 5 mM Tris and 20 mM NaCl at pH 8.0. The toxins or venom fractions were then added to the V_H_Hs at a threefold molar excess (V_H_H1 a-CTx: cardiotoxin (P01468), V_H_H5 a-sNTx: α-short-chain neurotoxin (P01426)) and incubated overnight at 4 °C. The V_H_H:toxin complexes were purified using size-exclusion chromatography (Superdex 75 10/300GL column, Cytiva) on an NGC Quest 10 Plus Chromatography system (Bio-Rad) maintained at 4 °C, with the reconstitution buffer serving as the mobile phase. Before crystal screening, the V_H_H–toxin complexes were concentrated to 15.0 mg ml^−1^ using 3.0 kDa MWCO ultracentrifugation filters (UFC500324, Merck).

Crystallization trials were performed at 21 °C via the sitting drop vapour diffusion method. Drops (0.3 µl) were set up at reservoir:protein ratios of 2:1, 1:1, or 1:2 in a 96-well drop format on SWISSCI MRC 2 well crystallization plates (JENA) using LMB, BCS, Index, and Structure screening solutions (Hampton Research). The wells were sealed with crystal clear tape and equilibrated against 50 µl of reservoir solution. The V_H_H1 a-CTx co-crystal formed in 0.2 M ammonium acetate, 0.1 M sodium acetate, pH 4.6, 30% w/v PEG4000. The V_H_H5 a-sNTx co-crystal formed in 0.2 M sodium chloride, 0.1 M sodium acetate, pH 4.6, 30% v/v 2-methyl-2,4-pentanediol (MPD). The developed co-crystals were collected using mounted CryoLoops (Hampton Research) with cryoprotection performed by adding glycerol to a neighbour drop with no crystals to a final concentration of 25%. The loop edge was kept in contact with the cryo solution for approximately 5 s to equilibrate before flash freezing the co-crystal in liquid nitrogen and shipping to the beamline for remote data collection. Data collection and refinement statistics are shown in Supplementary Tables 10 and 11. The final structural models and corresponding structure factors have been deposited in the Protein Data Bank (PDB) under accession codes 9RIT and 9RIU.

### Data collection and structure determination

X-ray diffraction data for the V_H_H1 a-CTx and V_H_H5 a-sNTx co-crystals were obtained at the Biomax (MAX IV synchrotron facility, Lund, Sweden) beamline. Complete datasets were collected over a 360° rotation for the V_H_H1 a-CTx and V_H_H5 a-sNTx co-crystals. The data processing was performed with XDSAPP3^75–77^, and the data are summarized in Supplementary Tables 10 and 11. Structures of the V_H_Hs in complex with their respective toxins were determined by molecular replacement with Phaser-MR^78^ using an AlphaFold 3 model for both the V_H_H and the target toxin as a search model. Model building and refinement were performed with Phenix.refine^77^ and Coot^79^.

The structures were evaluated using MolProbity with final statistics presented in Supplementary Tables 10 and 11. Molecular graphics were presented with PyMOL Molecular Graphics System (v.2.2r7pre, Schrödinger, LLC). Coordinates and structure factors have been submitted to the PDB database with the accession codes 9RIT and 9RIU.

### Cryo-EM collection and processing

Cryo grids of V_H_H20 a-PLA_2_ in complex with PLA_2_-3 were imaged at 190,000x nominal magnification using a Falcon 4i camera on a Glacios microscope at 200 kV. Automated image collection was performed using EPU from ThermoFisher. Images were aligned, dose-weighted, and Contrast Transfer Function (CTF)-corrected in the CryoSPARC Live software platform, with automated image collection also performed using Smart EPU software (ThermoFisher). Data processing was carried out in CryoSPARC v.4.5.3^80^. Blob particle picking was performed on all micrographs with a minimum particle diameter of 60 Å and a maximum of 90 Å. Particles extracted at 256 pixels box size were used to perform 2D classification, which were then used to generate a 3D reference model from ab initio refinement, followed by heterogeneous refinement and 3D classifications to obtain a good class that was further non-uniform heterogeneous refined. Gold-standard Fourier shell correlation resolution was calculated to be 5.4 Å. Owing to the small size of the complex and the low resolution of the map, we could not build a model, but could dock it in the AlphaFold 3 predicted complex as an indicator of whether the predicted interface is plausible.

### Generation of in silico predictions of V_H_H:toxin complexes

For V_H_H:toxin complexes that did not yield protein co-crystals, protein sequences were submitted as input to AlphaFold 3 for structure prediction^81^. Multiple predictions were generated using randomized seeds for each V_H_H:toxin complex. The model exhibiting the highest confidence scores (per-residue confidence estimate (pLDDT), predicted template modelling (pTM), and interface predicted template modelling (ipTM)) were selected for further analysis. Molecular visualization and graphic preparation were presented with PyMOL Molecular Graphics System (v.2.2r7pre, Schrödinger, LLC).

### In vivo neutralization of venom-induced lethality

LD_50_ determinations and lethality neutralization experiments were conducted using groups of mice of both sexes weighing 18–20 g. At IBt-UNAM, the CD1 mouse strain was used in the experiments performed for designing the recombinant antivenom, LD_50_ determinations, and rescue experiments. In experiments performed at the University of Northern Colorado, the NSA mouse strain was used for the pre-incubation assays. Time of death after administration of 3 × LD_50_ of venoms was recorded in both strains to secure homogeneous results. All mice were kept under 12 h light and dark cycles with food and water ad libitum, ambient temperature between 18 and 24 °C and relative humidity of approximately 60%. LD_50_s were determined for selected toxins (lNTx-7 and sNTx-3) and all the target venoms using the intravenous route (Supplementary Table 6) as described^20^. In the case of venoms selected for rescue assays, LD_50_s were also determined using the subcutaneous route (Supplementary Table 6).

#### Recombinant antivenom design experiments

To evaluate the neutralizing efficacy of the V_H_Hs and design a recombinant antivenom, neutralization of selected individual toxins and selected whole venoms was performed in pre-incubation experiments (Extended Data Fig. 4 and Supplementary Table 7), as described^20^. The mice were observed during the first 3 h and then approximately every 6 h for signs of envenoming. The percentage of survival was determined 24 h after the injection and plotted as Kaplan–Meier survival curves using GraphPad Prism v.10.2.

#### Pre-incubation experiments

For pre-incubation experiments of whole venoms, 3 × LD_50_ of each venom (Supplementary Table 6) were mixed with 3.6 mg (117 µl) of recombinant antivenom (Supplementary Table 8) in a total volume of 200 µl per mouse. This was then pre-incubated at 37 °C for 30 min before intravenous injection into groups of five mice. To compare the performance of the recombinant antivenom with a current plasma-derived commercial antivenom, five venoms were also tested for neutralization with the F(ab’)_2_ polyclonal antivenom Inoserp PAN-AFRICA (lot 5IT11003; expiration date November 2018) (INOSAN BioPharma), which is currently recommended for the treatment of envenomings caused by eight elapid and five viperid snakes from Africa. The antivenom was pre-incubated with the venom at 37 °C for 30 min, using the volume that neutralizes a minimum of 3 × LD_50_ of venom from *N. nigricollis* and *D. polylepis*, according to the manufacturer’s product insert. This antivenom is also recommended for the treatment of bites by the elapid snakes *D. viridis*,* D. angusticeps*,* D. jamesoni*, *N. haje*,* N. pallida*,* N. melanoleuca*,* N. nivea* and *N. katiensis*. All mice were observed during the first 5 h and then approximately every 6 h for appearance of envenoming signs (Supplementary Table 9). The percentage of survival was determined 24 h after the injection and plotted as Kaplan–Meier survival curves using GraphPad Prism v.10.2.

#### Rescue experiments

The venoms of 11 elapid snakes (*D. angusticeps*, *D. jamesoni*, *D. polylepis*, *D. viridis*, *N. annulifera*, *N. haje*, *N. melanoleuca*, *N. nivea*, *N. nubiae*, *N. senegalensis* and *H. haemachatus*) were selected for their neutralization in rescue experiments. These were designed to better represent actual envenoming, where the venom is injected first (subcutaneously) and then the recombinant antivenom is administered using the intravenous route. In these experiments, 3 × LD_50_ of each of the selected venoms (Supplementary Table 6) were injected in a final volume of 40 µl PBS. The recombinant antivenom was injected 5 min later using the intravenous route in a total volume of 300 µl PBS. Since the recombinant antivenom was designed considering the LD_50_ of each venom determined through intravenous administration, the dose of the recombinant antivenom used was adjusted based on the ratio between LD_50_s determined through subcutaneous and intravenous injection for each venom. The mice were observed during the first 5 h and then approximately every 6 h for the appearance of envenoming signs. The percentage of survival was determined 24 h after the injection and plotted as Kaplan–Meier survival curves using GraphPad Prism v.10.2.

To compare the performance of the recombinant antivenom with a current plasma-derived commercial antivenom, rescue experiments were performed for some species (*D. jamesoni*,* D. viridis*,* N. haje*,* N. melanoleuca* and *H. haemachatus*) using Inoserp PAN-AFRICA (lot 5IT11003; expiration date November 2018) (INOSAN BioPharma). Similar to the pre-incubation experiments, the antivenom dose was the volume that, according to the manufacturer, neutralizes a minimum of 3 × LD_50_ of venom adjusted based on the ratio between the LD_50_ determined by intravenous or subcutaneous injection.

Owing to the low availability of commercial antivenom, a vial from an expired batch of Inoserp PAN-AFRICA was used for all experiments.

### In vivo neutralization of venom-induced dermonecrosis

For dermonecrosis experiments, groups (*n* ≥ 5) of male Swiss (CD1) mice (29–31 g) were used. Animals had ad libitum access to CRM-irradiated food and filtered water. Prior to venom injection, mice were weighed and given 5 mg kg^−1^ subcutaneous morphine. The dorsal flanks of mice were shaved to monitor lesion progression.

#### Prevention of venom-induced dermonecrosis

For venom challenges, mice were injected intradermally in the ventral abdominal region, with venoms from *N. nigricollis* (24 µg per mouse), *N. mossambica* (39 µg per mouse) and *H. haemachatus* (26 µg per mouse) dissolved in 50 µl PBS. This dose corresponds to 1 minimum necrotizing dose (MND)—that is, the dose that induces an area of dermonecrosis of 5 mm in diameter, 72 h after injection^38^. In pre-incubation models, 1 MND of venom from each of the 3 snakes was pre-incubated with 1.09 mg of a mixture of V_H_H1 a-CTx (450 µg per mouse), V_H_H4 a-CTx (450 µg per mouse) and V_H_H20 a-PLA_2_ (190 µg per mouse) at 37 °C for 30 min before intradermal injection. In the first rescue model, 1 MND dose of venom in 10 µl was injected intradermally, followed by 1.09 mg of V_H_Hs in 40 µl at the same region after 15 min. In a second rescue model, 1 MND dose of venom was injected intradermally in a 50 µl volume, followed after 15 min by intravenous administration of 3.6 mg of recombinant antivenom (Supplementary Table 8) in 200 µl. For the control groups the same volume of PBS was administered instead of V_H_Hs. As a comparison, a group of mice received 1 MND of *N. nigricollis* venom followed by 4.2 mg of Inoserp PAN-AFRICA antivenom (INOSAN Biopharma).

Mice were monitored continuously for the first 6 h post-injection, with additional checks every 3 h up to 12 h and then 3 times daily up to 72 h in pre-incubation and intradermal rescue models and up to 48 h in intravenous rescue studies. At the end of each experiment, animals were humanely euthanized via inhalational CO_2_. Lesions at injection sites were dissected, measured in two directions with digital callipers, and photographed with a camera and light ring.

### Statistical analysis

To evaluate the significance of outcomes from these experiments, a Welch’s *t*-test was used to compare mean lesion sizes between control and treatment groups, following confirmation that data met parametric assumptions. Normality was verified using the Shapiro–Wilk test, while ROUT tests were performed to identify any outliers within the data. Comparisons were made against the negative control (PBS only). All analyses were conducted using GraphPad Prism (v.10.3.1), with statistical significance set at *α* = 0.05.

### Ethics declarations

For systemic envenoming experiments, all animals and in vivo methodologies used were approved by the bioethics committee of the Institute of Biotechnology, Universidad Nacional Autónoma de México (IBt-UNAM) under project 410 or the University of Northern Colorado Institutional Animal Care and Use Committee (UNC-IACUC), the Department of Biological Sciences under project 2208D-SM-SMLBirds. For dermonecrosis experiments, ethical approvals were obtained from the Animal Welfare and Ethics Review Boards of Liverpool School of Tropical Medicine and The University of Liverpool, and work was performed under UK Home Office Project Licences P58464F90 and PP2669304 in accordance with the UK Animal (Scientific Procedures) Act 1986.

### Reporting summary

Further information on research design is available in the Nature Portfolio Reporting Summary linked to this article.

## Online content

Any methods, additional references, Nature Portfolio reporting summaries, source data, extended data, supplementary information, acknowledgements, peer review information; details of author contributions and competing interests; and statements of data and code availability are available at 10.1038/s41586-025-09661-0.

## Supplementary information

Supplementary Table 1 **Venom fractions**. An overview of the snake species abbreviations, fraction names, toxin FASTA sequences, and content of the venom fractions used for phage display selections and screening of V_H_Hs. Key toxins used for labelling each fraction are highlighted in bold

Reporting Summary

Supplementary Tables 2–11 This file contains an array of supplementary tables describing the venom fractions, the camelid immunization scheme, the phage display campaigns, sequences of lead clones, fitting parameters of kinetic data, LD_50_s of used toxins and venoms, recombinant antivenom composition, in vivo experiments and refinement statistics of the co-crystal structures

## Data Availability

All the data supporting the present manuscript are available in the form of Source Data files and in the supplementary material. Relevant V_H_H and toxin sequences as well as detailed information on in vivo experiments are provided in the Supplementary Material. Raw data and analyses performed for the figures are available as Source Data Files. Final structural models and corresponding structure factors have been deposited in the Protein Data Bank (PDB) under accession codes: 9RIT and 9RIU. The EM map has been deposited at the Electron Microscopy Data Bank (EMDB) with the accession code EMD-73038. Proteins with the following accession numbers were used in the study: A8N285, C0HJB0, C0HJD7, P00600, P00605, P00979, P00981, P00984, P00986, P01388, P01389, P01390, P01391, P01400, P01405, P01407, P01416, P01417, P01419, P01421, P01422, P01423, P01424, P01431, P01433, P01448, P01452, P01456, P01457, P01462, P01463, P01468, P01473, P01419, P01477, P01478, P01484, P01485, P01486, P01487, P01488, P01489, P01490, P01491, P01492, P01493, P01494, P01495, P01496, P01497, P01498, P01499, P14556, P17696, P18328, P18329, P24777, P24778, P25517, P25678, P25682, P25683, P25687, P60237, P62394, P68418, P82462, P0DQP2, P0DQQ2, P0DSN1, Q53B57 and Q9YGJ6.
