# Supplementary material for: Nanobody-based recombinant antivenom for cobra, mamba and rinkhals bites
Source: Nature. 2025 Oct 29;647(8090):716–25. doi: 10.1038/s41586-025-09661-0 (PMC12629983; doi:10.1038/s41586-025-09661-0)
Supplement: Supplementary file 2 — Reporting Summary [file 41586_2025_9661_MOESM2_ESM.pdf]

nature portfolio

Corresponding author(s): Timothy P. Jenkins
Anne Ljungars
Andreas H. Laustsen

Last updated by author(s): Sep 8, 2025

Reporting Summary

Nature Portfolio wishes to improve the reproducibility of the work that we publish. This form provides structure for consistency and transparency in reporting. For further information on Nature Portfolio policies, see our Editorial Policies and the Editorial Policy Checklist.

Statistics

For all statistical analyses, confirm that the following items are present in the figure legend, table legend, main text, or Methods section.

n/a	Confirmed	
☐	☒	The exact sample size (*n*) for each experimental group/condition, given as a discrete number and unit of measurement
☐	☒	A statement on whether measurements were taken from distinct samples or whether the same sample was measured repeatedly
☐	☒	The statistical test(s) used AND whether they are one- or two-sided<br>*Only common tests should be described solely by name; describe more complex techniques in the Methods section.*
☒	☐	A description of all covariates tested
☐	☒	A description of any assumptions or corrections, such as tests of normality and adjustment for multiple comparisons
☐	☒	A full description of the statistical parameters including central tendency (e.g. means) or other basic estimates (e.g. regression coefficient) AND variation (e.g. standard deviation) or associated estimates of uncertainty (e.g. confidence intervals)
☐	☒	For null hypothesis testing, the test statistic (e.g. *F*, *t*, *r*) with confidence intervals, effect sizes, degrees of freedom and *P* value noted<br>*Give P values as exact values whenever suitable.*
☒	☐	For Bayesian analysis, information on the choice of priors and Markov chain Monte Carlo settings
☒	☐	For hierarchical and complex designs, identification of the appropriate level for tests and full reporting of outcomes
☒	☐	Estimates of effect sizes (e.g. Cohen's *d*, Pearson's *r*), indicating how they were calculated

Our web collection on statistics for biologists contains articles on many of the points above.

Software and code

Policy information about availability of computer code

Data collection	Patch Clamp data was collected with Sophion ViewPoint v6.6.70 (Sophion Bioscience). BLI data was collected using Octet" Analysis Studio 12.2.2.26 (ForteBio). DELFIA data was collected using VICTOR Nivo® Control software v5.1.0.
Data analysis	Patch Clamp data was analyzed with Sophion Analyzer v6.6.70 {Sophion Bioscience) and Graph Pad Prism vl0. BLI data was processed and analyzed using Octet® Analysis Studio 12.2.2.26 (ForteBio) and GraphPad Prism vl0. cryoEM images were analysed in the CryoSPARC LiveTM software platform, and Smart EPU software (ThermoFisher). cryoEM data processing was carried out using the software CryoSPARC v4.5.3. All other data analysis was performed on GraphPad Prism v10.

For manuscripts utilizing custom algorithms or software that are central to the research but not yet described in published literature, software must be made available to editors and reviewers. We strongly encourage code deposition in a community repository (e.g. GitHub). See the Nature Portfolio guidelines for submitting code & software for further information.

Data

Policy information about availability of data

All manuscripts must include a data availability statement. This statement should provide the following information, where applicable:

- Accession codes, unique identifiers, or web links for publicly available datasets
- A description of any restrictions on data availability
- For clinical datasets or third party data, please ensure that the statement adheres to our policy

All the data supporting the present manuscript is available in the form of Source Data Files and in the supplementary material. Relevant nanobody and toxin sequences as well as detailed information on in vivo experiments are provided in the Supplementary Material. Raw data and analyses performed for the figures are available as Source Data Files. Final structural models and corresponding structure factors have been deposited in the Protein Data Bank (PDB) under accession codes: 9RIT and 9RIU. Proteins with the following accession numbers were used in the study: A8N285, C0HJB0, C0HJD7, P00600, P00605, P00979, P00981, P00984, P00986, P01388, P01389, P01390, P01391, P01400, P01405, P01407, P01416, P01417, P01419, P01421, P01422, P01423, P01424, P01431, P01433, P01448, P01452, P01456, P01457, P01462, P01463, P01468, P01473, P01419, P01477, P01478, P01484, P01485, P01486, P01487, P01488, P01489, P01490, P01491, P01492, P01493, P01494, P01495, P01496, P01497, P01498, P01499, P14556, P17696, P18328, P18329, P24777, P24778, P25517, P25678, P25682, P25683, P25687, P60237, P62394, P68418, P82462, P0DQP2, P0DQQ2, P0DSN1, Q53B57, and Q9YGJ6.

Research involving human participants, their data, or biological material

Policy information about studies with human participants or human data. See also policy information about sex, gender (identity/presentation), and sexual orientation and race, ethnicity and racism.

Reporting on sex and gender	n/a
Reporting on race, ethnicity, or other socially relevant groupings	n/a
Population characteristics	n/a
Recruitment	n/a
Ethics oversight	n/a

Note that full information on the approval of the study protocol must also be provided in the manuscript.

Field-specific reporting

Please select the one below that is the best fit for your research. If you are not sure, read the appropriate sections before making your selection.

☒ Life sciences ☐ Behavioural & social sciences ☐ Ecological, evolutionary & environmental sciences

For a reference copy of the document with all sections, see nature.com/documents/nr-reporting-summary-flat.pdf

Life sciences study design

All studies must disclose on these points even when the disclosure is negative.

Sample size	Samples sizes were selected according to standard protocols for each methodology.<br>For in vivo assays, the smallest number of animals that ensured reliable results was used (3 to 5). According to: Lorke, D. (1983). A new approach to practical acute toxicity testing. Archives of Toxicology, 54(4), 275–287. https://doi.org/10.1007/BF01234480<br>For electrophysiology, 16 individual cells per treatment were run in 384 well plates.
Data exclusions	Cells which did not reach a proper patch clamp seal were excluded since no reliable data can be obtained from them.
Replication	Replicates were successful. For in vivo data, single experiments with 3 or 5 mice per group were done. All controls were done in separate days compared to the treatments. Enzymatic PLA2 assay replicates were performed in different dates in duplicates. Electrophysiology experiments were performed at the same time with cells patched individually in 384 well plates, but in different dates for different venoms fractions, toxins, venoms, or nanobodies.
Randomization	No randomisation method was used to allocate experimental units to treatment groups and potential confounders were not controlled for. The experimental unit was one cage of five animals, and animals were randomised into cages of five upon arrival. One experimental unit (one cage of five mice) was allocated to each treatment group.
Blinding	All experimenters were unblinded throughout the experiment and analyses.

Reporting for specific materials, systems and methods

We require information from authors about some types of materials, experimental systems and methods used in many studies. Here, indicate whether each material, system or method listed is relevant to your study. If you are not sure if a list item applies to your research, read the appropriate section before selecting a response.

Materials & experimental systems

n/a	Involved in the study	
☐	☒	Antibodies
☐	☒	Eukaryotic cell lines
☒	☐	Palaeontology and archaeology
☐	☒	Animals and other organisms
☒	☐	Clinical data
☒	☐	Dual use research of concern
☒	☐	Plants

Methods

n/a	Involved in the study	
☒	☐	ChIP-seq
☒	☐	Flow cytometry
☒	☐	MRI-based neuroimaging

Antibodies

Antibodies used	The nanobody phage display library used in this study was prepared for the Center for Antibody Technologies from two camelids immunized with 18 elapid snake venoms at the VIB Nanobody Core (Brussels, Belgium). The following commercial antibodies were used: anti-FLAG antibody clone M2 (F3165,Sigma-Aldrich).
Validation	From manufacturer's websites: Anti-FLAG M2: Monoclonal ANTI-FLAG® M2 is a purified immunoglobulin, lgGl, monoclonal antibody, isolated from murine ascites fluid, that binds to FLAG® fusion proteins. The M2 antibody will recognize the FLAG" sequence at the N-terminus, Met-N-terminus, (-terminus, or at an internal site of FLAG® fusion proteins. Monoclonal ANTI-FLAG® M2 is useful for identification and capture of FLAG® fusion proteins by common immunological procedures such as Western blots and immuno-precipitation. It is also useful for affinity purification of FLAG" fusion proteins when bound to a solid support. Monoclonal ANTI-FLAG" M2 binding is not dependent on calcium.

Eukaryotic cell lines

Policy information about cell lines and Sex and Gender in Research

Cell line source(s)	Rhabdomyosarcoma (RD) cells, CCL-136TM from the American Type Culture Collection (ACTT). Origin: Homo sapiens, human, Female. Non-commercial human N/TERT immortalized keratinocytes.
Authentication	qPCR of nACh receptor to validate correct ion channel target
Mycoplasma contamination	Tested negative for Mycoplasma contamination
Commonly misidentified lines (See ICLAC register)	Rhabdomyosarcoma (RD) cells, CCL-136:<br>Misidentified Cell Line: TE671; Registration ID: ICLAC-00101; Claimed Species: Human; Claimed Cell Type: Medulloblastoma; Misidentified Cell Line, Cellosaurus AC: CVCL_1756; Contaminating Cell Line: RD; Actual Species: Human; Actual Cell Type: Sarcoma (rhabdomyosarcoma); Contaminating Cell Line, Cellosaurus AC: CVCL_1649; Misidentification Reported By: Stratton et al, 1989; Chen et al, 1989; Reference PubMed ID: 2650908, 2739733<br>Rationale for use: Validated expression of the relevant nACh receptor<br>Human N/TERT immortalized keratinocytes were kindly donated by Edel O'Toole from the Queen Mary University of London.

Animals and other research organisms

Policy information about studies involving animals; ARRIVE guidelines recommended for reporting animal research, and Sex and Gender in Research

Laboratory animals	Animal experiments were conducted in CD-1 or NSA mice of both sexes (corresponding to 4-5 weeks old). Mice were supplied by the animal facility of the Instituto de Biotecnologia, Universidad Nacional Autonoma de Mexico (IBt-UNAM), Cuernavaca, Mexico, from the LSTM and University of Liverpool AWERBs and the UK Home Office, or the University of Northern Colorado (UNC). Mice were provided food and water ad libitum and housed in standard cages in groups of maximum 5 mice per cage.<br>Animals were maintained at 18-24 C, relative humidity of approximately 60% and 12:12 light-dark cycle.
Wild animals	No wild animals were used in the study
Reporting on sex	Mice of both sexes were used in the study
Field-collected samples	No field collected samples were used in this study

Ethics oversight	For systemic envenoming experiments, all animals and in vivo methodologies used were approved by the bioethics committee of the Institute of Biotechnology, Universidad Nacional Autónoma de México (IBt-UNAM) under project 410 or the University of Northern Colorado Institutional Animal Care and Use Committee (UNC-IACUC), the Department of Biological Sciences under project 2208D-SM-SMLBirds. For dermonecrosis experiments, ethical approvals were obtained from the Animal Welfare and Ethics Review Boards of Liverpool School of Tropical Medicine and The University of Liverpool, and work was performed under UK Home Office Project Licences P58464F90 and PP2669304 in accordance with the UK Animal (Scientific Procedures) Act 1986.

Note that full information on the approval of the study protocol must also be provided in the manuscript.

Plants

Seed stocks	*Report on the source of all seed stocks or other plant material used. If applicable, state the seed stock centre and catalogue number. If plant specimens were collected from the field, describe the collection location, date and sampling procedures.*
Novel plant genotypes	*Describe the methods by which all novel plant genotypes were produced. This includes those generated by transgenic approaches, gene editing, chemical/radiation-based mutagenesis and hybridization. For transgenic lines, describe the transformation method, the number of independent lines analyzed and the generation upon which experiments were performed. For gene-edited lines, describe the editor used, the endogenous sequence targeted for editing, the targeting guide RNA sequence (if applicable) and how the editor was applied.*
Authentication	*Describe any authentication procedures for each seed stock used or novel genotype generated. Describe any experiments used to assess the effect of a mutation and, where applicable, how potential secondary effects (e.g. second site T-DNA insertions, mosiacism, off-target gene editing) were examined.*
