# Supplementary material for: Nanobody-based recombinant antivenom for cobra, mamba and rinkhals bites
Source: Nature. 2025 Oct 29;647(8090):716–25. doi: 10.1038/s41586-025-09661-0 (PMC12629983; doi:10.1038/s41586-025-09661-0)
Supplement: Supplementary file 3 — This file contains an array of supplementary tables describing the venom fractions, the camelid immunization scheme, the phage display campaigns, sequences of lead clones, fitting parameters of kinetic data, LD50s of used toxins and venoms, recombinant antivenom composition, in vivo experiments and refinement statistics of the co-crystal structures [file 41586_2025_9661_MOESM3_ESM.pdf]

Supplementary information

Nanobody-based recombinant antivenom for cobra, mamba, and rinkhals bites

Shirin Ahmadi[1,10], Nick J. Burlet[1,10], Melisa Benard-Valle[1], Alid Guadarrama-Martínez[2], Samuel Kerwin[3], Iara A. Cardoso[4,5], Amy E. Marriott[6], Rebecca J. Edge[6], Edouard Crittenden[4], Edgar Neri-Castro[2], Monica L. Fernandez-Quintero[7], Giang T. T. Nguyen[1], Carol O'Brien[1], Yessica Wouters[1], Konstantinos Kalogeropoulos[1], Suthimon Thumtecho[1], Tasja Wainani Ebersole[1], Camilla Holst Dahl[1], Emily U. Glegg-Sørensen[1], Tom Jansen[1], Kim Boddum[8], Evangelia Manousaki[1], Esperanza Rivera-de-Torre[1], Andrew B. Ward[7], J. Preben Morth[1], Alejandro Alagón[2], Stephen P. Mackessy[3], Stuart Ainsworth[6], Stefanie K. Menzies[9], Nicholas R. Casewell[4], Timothy P. Jenkins[1#], Anne Ljungars[1#], Andreas H. Laustsen[1#]

[1]Department of Biotechnology and Biomedicine, Technical University of Denmark, DK-2800 Kongens Lyngby, Denmark

[2]Departamento de Medicina Molecular y Bioprocesos, Instituto de Biotecnología, Universidad Nacional Autónoma de México, Avenida Universidad 2001, Cuernavaca, Mor., 62210, México

[3]Department of Biological Sciences, University of Northern Colorado, 501 20th St., CB 92, Greeley, CO 80639-0017 USA

[4]Centre for Snakebite Research and Interventions, Department of Tropical Disease Biology, Liverpool School of Tropical Medicine, Pembroke Place, Liverpool, L3 5QA, United Kingdom

[5]School of Biochemistry, University of Bristol, Biomedical Sciences Building, University Walk, Bristol, BS8 1TD, United Kingdom

[6]Department of Infection Biology and Microbiomes, Institute of Infection, Veterinary and Ecological Sciences, University of Liverpool, Liverpool, L3 5RF, United Kingdom

[7]Department of Integrative Structural and Computational Biology, The Scripps Research Institute, La Jolla, CA, USA

[8]Sophion Bioscience, DK-2750 Ballerup, Denmark

[9]Biomedical & Life Sciences, Faculty of Health and Medicine, Lancaster University, Lancaster, United Kingdom LA1 4YG

[10]Equal contributions

[#]Corresponding authors: Timothy P. Jenkins tpaje@dtu.dk, Anne Ljungars aellj@dtu.dk, and Andreas H. Laustsen ahola@bio.dtu.dk

Supplementary Table 1. Snake species abbreviations, fraction names, toxin FASTA sequences, and content of the venom fractions used for phage display selection and screening of V_HHs. Key toxins used for labeling each fraction are highlighted in bold. The toxins used for labeling of the fractions were based on the clinically most relevant one.

To facilitate reading, this table is included as an Excel file.

Supplementary Table 2. Immunization scheme for each camelid animal, including the time points for phage display library construction.

Day/week	Total venom dose (mg)	Dose per venom (mg)	Library generation
0 d	0.27	0.015	No
14 d	0.54	0.03	No
28 d	0.72	0.04	No
42 d	0.99	0.06	No
46 d	-	-	Yes (Library A)
49 d	-	-	
56 d	1.26	0.07	No
70 d	1.44	0.08	No
84 d	1.80	0.10	No
98 d	2.16	0.12	No
102 d	-	-	Yes (Library B)
105 d	-	-	
52 w	4.5	0.25	No
54 w	4.5	0.25	No
60 w	4.5	0.25	No
60 w + 5 d			Yes (Library C)
60 w + 8 d			

Supplementary Table 3. Overview of the phage display campaigns. Toxin (sub)family, library, venom fraction or toxin, and the target toxin concentrations have been included for each round of panning. The labels for phage pool outputs selected for screening and the V_HHs characterized from each selection are shown in the last 2 columns.

**Toxin (sub)family**	**Round 1**			**Round 2**		**Round 3**		**Selection number**	**Characterized V_HHs**
	**Library**	**Target**	**Concentration (nM)**	**Target**	**Concentration (nM)**	**Target**	**Concentration (nM)**		
CTx	A + B	CTx-10	50	CTx-7	100	CTx-10	50	TPL0870	V_HH1, V_HH2, V_HH3
	C	CTx-12	100	CTx-11	100	-	-	TPL1054	V_HH4
sNTx	C	sNTx-1	100	sNTx-1	100	sNTx-1	10	TPL0998	V_HH5, V_HH6
						sNTx-3	10	TPL1000	V_HH7
						sNTx-6	10	TPL1006	V_HH8
lNTx	A + B	lNTx-1	50	lNTx-1	50	lNTx-1	50	TPL0830	V_HH12
						lNTx-3	5	TPL0833	V_HH10, V_HH11
	A + B	lNTx-7	100	lNTx-7	50	lNTx-3	50	TPL0877	V_HH9
AgTx	C	AgTx-2	100	AgTx-2	100	AgTx-2	1	TPL1033	V_HH13, V_HH14
Og XI	C	Og XI-1	100	Og XI-1	100	Og XI-1	10	TPL1019	V_HH16
	C	Og XI-2	100	Og XI-2	100	Og XI-2	10	TPL1039	V_HH15
PLA_2	B	PLA_2-3	50	PLA_2-3	50	-	-	TPL0599	V_HH19, V_HH20
					5	-	-	TPL0615	V_HH21
KUN	C	KUN-1	100	KUN-1	100	KUN-1	10	TPL1011	V_HH18
						KUN-2	10	TPL1013	V_HH17, V_HH19

Supplementary Table 4. Sequence of CDR3 (hit clones) or whole sequence (lead clones) for included V_HHs.

V_HH	Target	Amino acid sequence
V_HH1 a-CTx	CTx	QVQLQESGGGLVQAGGSLRLSCAASGRTFSSYAMAWFRQAPGKEREFVASISWNGDSTYYADSVKGRFTISGDNAKNTWYLQMKSLKPEDTAVYYCNTEDEGSGTYYEWGQGTQVTVSSAAADYKDHDGDYKDHDIDYKDDDDKGAAHHHHHH
V_HH4 a-CTx	CTx	QVQLQESGGGLVQAGGSLRLSCAAPGFTFDNYAIGWFRQAPGKEREAVSMIDVNDGDTYYADSVKGRFATSIDNARITAYLQMNSLKPEDTAVYYCAAGEQGGIETEDDYGMDYYGKGTQVTVSSAAADYKDHDGDYKDHDIDYKDDDDKGAAHHHHHH
V_HH5 a-sNTx	sNTx	QVQLQESGGGLVRPGGSLRLSCAVSGVSESIFANYVVGWFRQQDSGTGRDLVAQSSSDDEYNHVSGSVKGRFTISRDNAKNTVSLQMDNLKPEDTAIYICAAAIGPGDNYIYWGQGTQVTVSSAAADYKDHDGDYKDHDIDYKDDDDKGAAHHHHHH
V_HH9 a-lNTx	lNTx	QVQLQESGGGLVQPGGSLRLSCTASGSIFSTVNTMGWYRQAPGKQRELVAAITGGGSTNYADSVKGRFTISRDNVENTVHLRMNALKPEDTAVYYCNFQEYCSAYGCYELPHDYWGQGTQVTVSSAAADYKDHDGDYKDHDIDYKDDDDKGAAHHHHHH
V_HH13 a-AgTx	AgTx	QVQLQESGGGLVQAGGSLRLSCAASGLDFSTMPLTVGWFRQAPGKEREFVAAISWRGDSWNYEDGMEGRFTVSRDNAKNTVYLQMSSLHTEDTGVYYCAGDPTPYGTSSGGFSVWGQGTQVTVSSAAADYKDHDGDYKDHDIDYKDDDDKGAAHHHHHH
V_HH15 a-Og XI	Og XI	QVQLQESGGGAVPAGGSLTLSCAASGRTLSDYTMGWFRQAPGKEREIVGAISWNDGYTYYDDSVKGRFTISRDNAKNTMYLQMNSLKPEDTAVYYCAAQAPEMVRSGNFGSWGPGTQVTVSSAAADYKDHDGDYKDHDIDYKDDDDKGAAHHHHHH
V_HH17 a-KUN	KUN	QVQLQESGGGLVQPGGSLRLSCITSGSNFDDLSVGWFRQAPGKEREGVSCFSSSDGSTYYVDSVKGRFTMSRDNAKNTVYLQMNNLKPEDTAVYYCTADGFPYPSETMCSIPGGPDSVAWGQGTQVTVSSAAADYKDHDGDYKDHDIDYKDDDDKGAAHHHHHH
V_HH20 a-PLA_2	PLA_2	QVQLQESGGGLAQAGGSLRLSCAASGLAFSNYSMGWARQAPGKEREFVAAISWSHQPTYYADSVKGRFTISRDNAKNTLYLQMNSLKPEDTAVYYCAADSRPNSYRLGGPGNYWGQGTQVTVSSAAADYKDHDGDYKDHDIDYKDDDDKGAAHHHHHH

V_HH	Target	Amino acid sequence CDR3
V_HH2 a-CTx	CTx	AANEPEDCSGSACDGGS
V_HH3 a-CTx	CTx	SPDYGLVDTASCEYGY
V_HH6 a-sNTx	sNTx	AAALGPGDNYIYY
V_HH7 a-sNTx	sNTx	AAAPGDQYTN
V_HH8 a-sNTx	sNTx	AAALGPGDLIYYY
V_HH10 a-lNTx	lNTx	NADSYCSDSVCYENPVYDY
V_HH11 a-lNTx	lNTx	AAERVPSAIYTHYEFCVGADEYDY
V_HH12 a-lNTx	lNTx	NAAGVVYDTGVYMDYTKDY
V_HH14 a-AgTx	AgTx	NSDLYTEYGQMWDL
V_HH16 a-Og XI	Og XI	AAQSPDMVRVGNFGS
V_HH18 a-KUN	KUN	EGYDGSHYIDY
V_HH19 a-KUN	KUN	TADGFPYPSETMCSIPGGPDSVA
V_HH21 a-PLA_2	PLA_2	NLHRLTSSDDDGRT

Supplementary Table 5. Affinity measurements between V_HHs and venom fractions or toxins using BLI. x: no binding.

Toxin (sub)family	Clone	Target	K_D (M)	K_D error	K_{on} (1/Ms)	k_{on} error	K_{off} (1/s)	k_{off} error	R^2
CTx	V_HH1 a-CTx	CTx-1	x	x	x	x	x	x	x
		CTx-10	$3.28\cdot10^{-9}$	$5.23\cdot10^{-11}$	$7.42\cdot10^{4}$	$5.10\cdot10^{2}$	$2.44\cdot10^{-4}$	$3.51\cdot10^{-6}$	0.99
		CTx-12	$1.91\cdot10^{-7}$	$8.76\cdot10^{-9}$	$2.31\cdot10^{4}$	$1.00\cdot10^{3}$	$4.40\cdot10^{-3}$	$6.68\cdot10^{-5}$	0.9191
		CTx-13	x	x	x	x	x	x	x
		CTx-18	$8.22\cdot10^{-9}$	$1.03\cdot10^{-10}$	$5.64\cdot10^{4}$	$4.55\cdot10^{2}$	$4.64\cdot10^{-4}$	$4.47\cdot10^{-6}$	0.9878
	V_HH2 a-CTx	CTx-10	$3.81\cdot10^{-9}$	$4.28\cdot10^{-11}$	$1.47\cdot10^{5}$	$1.25\cdot10^{3}$	$5.61\cdot10^{4}$	$4.15\cdot10^{-6}$	0.9825
		CTx-18	$9.42\cdot10^{-9}$	$1.02\cdot10^{-10}$	$1.13\cdot10^{5}$	$1.09\cdot10^{3}$	$1.07\cdot10^{-3}$	$5.41\cdot10^{-6}$	0.9837
	V_HH4 a-CTx	CTx-1	x	x	x	x	x	x	x
		CTx-10	x	x	x	x	x	x	x
		CTx-12	$6.64\cdot10^{-10}$	$3.57\cdot10^{-11}$	$1.02\cdot10^{5}$	$7.73\cdot10^{2}$	$6.74\cdot10^{-5}$	$3.58\cdot10^{-6}$	0.9858
		CTx-13	$3.20\cdot10^{-10}$	$5.92\cdot10^{-12}$	$4.18\cdot10^{5}$	2.42·10+03	$1.34\cdot10^{-4}$	$2.35\cdot10^{-6}$	0.9854
		CTx-18	x	x	x	x	x	x	x
sNTx	V_HH5 a-sNTx	sNTx-1	$1.90\cdot10^{-9}$	$1.41\cdot10^{-10}$	$2.49\cdot10^{4}$	$1.49\cdot10^{2}$	$4.72\cdot10^{-5}$	$3.48\cdot10^{-6}$	0.9956
		sNTx-3	$2.88\cdot10^{-8}$	$2.20\cdot10^{-10}$	$2.35\cdot10^{4}$	$1.28\cdot10^{2}$	$6.77\cdot10^{-4}$	$3.61\cdot10^{-6}$	0.9965
		sNTx-5	$1.69\cdot10^{-8}$	$1.36\cdot10^{-10}$	$3.74\cdot10^{4}$	$2.15\cdot10^{2}$	$6.31\cdot10^{-4}$	$3.58\cdot10^{-6}$	0.9966
		sNTx-6	$5.46\cdot10^{-9}$	$8.11\cdot10^{-11}$	$2.98\cdot10^{4}$	$1.20\cdot10^{2}$	$1.63\cdot10^{-4}$	$2.33\cdot10^{-6}$	0.9981
		sNTx-7	$3.88\cdot10^{-10}$	$7.42\cdot10^{-11}$	$3.71\cdot10^{4}$	$1.85\cdot10^{2}$	$1.44\cdot10^{-5}$	$2.75\cdot10^{-6}$	0.9978
		sNTx-8	$<1.0\cdot10^{-12}$	$1.16\cdot10^{-10}$	$2.24\cdot10^{4}$	$9.40\cdot10^{1}$	$<1.0\cdot10^{-7}$	-	0.9981
		sNTx-9	$1.70\cdot10^{-9}$	$7.32\cdot10^{-11}$	$3.96\cdot10^{4}$	$2.11\cdot10^{2}$	$6.75\cdot10^{-5}$	$2.88\cdot10^{-6}$	0.9963
	V_HH6 a-sNTx	sNTx-1	$9.19\cdot10^{-10}$	$8.91\cdot10^{-11}$	$2.98\cdot10^{4}$	$1.38\cdot10^{2}$	$2.74\cdot10^{-5}$	$2.65\cdot10^{-6}$	0.9976
		sNTx-3	$6.24\cdot10^{-8}$	$4.27\cdot10^{-10}$	$2.56\cdot10^{4}$	$1.56\cdot10^{2}$	$1.60\cdot10^{-3}$	$4.97\cdot10^{-6}$	0.9966
		sNTx-8	$1.36\cdot10^{-9}$	$1.84\cdot10^{-10}$	$2.37\cdot10^{4}$	$1.89\cdot10^{2}$	$3.21\cdot10^{-5}$	$4.34\cdot10^{-6}$	0.989
	V_HH7 a-sNTx	sNTx-1	$4.55\cdot10^{-9}$	$7.39\cdot10^{-11}$	$5.01\cdot10^{4}$	$3.35\cdot10^{2}$	$2.28\cdot10^{-4}$	$3.37\cdot10^{-6}$	0.9967
		sNTx-3	$<1.0\cdot10^{-12}$	$6.03\cdot10^{-11}$	$8.07\cdot10^{4}$	$7.91\cdot10^{2}$	$<1.0\cdot10^{-7}$	-	0.9825
		sNTx-8	$3.37\cdot10^{-8}$	$3.01\cdot10^{-10}$	$5.24\cdot10^{4}$	$4.29\cdot10^{2}$	$1.77\cdot10^{-3}$	$6.28\cdot10^{-6}$	0.9923
		sNTx-9	$1.79\cdot10^{-8}$	$1.24\cdot10^{-10}$	$7.45\cdot10^{4}$	$4.60\cdot10^{2}$	$1.33\cdot10^{-3}$	$4.20\cdot10^{-6}$	0.9963
lNTx	V_HH9 a-lNTx	lNTx-3	$1.18\cdot10^{-9}$	$1.50\cdot10^{-11}$	$1.73\cdot10^{5}$	$8.88\cdot10^{2}$	$2.03\cdot10^{-4}$	$2.38\cdot10^{-6}$	0.9921
		lNTx-7	$7.53\cdot10^{-10}$	$2.51\cdot10^{-11}$	$3.60\cdot10^{4}$	$5.75\cdot10^{1}$	$2.71\cdot10^{-5}$	$9.03\cdot10^{-7}$	0.9998
	V_HH12 a-lNTx	lNTx-3	$1.47\cdot10^{-8}$	$8.76\cdot10^{-11}$	$8.30\cdot10^{4}$	$4.41\cdot10^{2}$	$1.22\cdot10^{-3}$	$3.32\cdot10^{-6}$	0.9953
AgTx	V_HH13 a-AgTx	AgTx-1	$7.16\cdot10^{-9}$	$9.88\cdot10^{-11}$	$4.91\cdot10^{4}$	$3.60\cdot10^{2}$	$3.52\cdot10^{-4}$	$4.12\cdot10^{-6}$	0.9946
		AgTx-2	$2.16\cdot10^{-9}$	$6.67\cdot10^{-11}$	$7.47\cdot10^{4}$	$6.68\cdot10^{2}$	$1.61\cdot10^{-4}$	$4.77\cdot10^{-6}$	0.99
	V_HH14 a-AgTx	AgTx-1	$7.02\cdot10^{-10}$	$5.93\cdot10^{-11}$	$5.87\cdot10^{4}$	$3.79\cdot10^{2}$	$4.12\cdot10^{-5}$	$3.47\cdot10^{-6}$	0.9954
		AgTx-2	$7.47\cdot10^{-9}$	$1.84\cdot10^{-10}$	$4.27\cdot10^{4}$	$5.26\cdot10^{2}$	$3.19\cdot10^{-4}$	$6.79\cdot10^{-6}$	0.9747
Og XI	V_HH15 a-Og XI	Og XI-1	$2.19\cdot10^{-9}$	$4.10\cdot10^{-11}$	$7.34\cdot10^{4}$	$3.95\cdot10^{2}$	$1.61\cdot10^{-4}$	$2.88\cdot10^{-6}$	0.9963
		Og XI-2	$2.16\cdot10^{-9}$	$5.42\cdot10^{-11}$	$5.67\cdot10^{4}$	$3.08\cdot10^{2}$	$1.22\cdot10^{-4}$	$3.00\cdot10^{-6}$	0.9972
	V_HH16 a-Og XI	Og XI-1	$2.40\cdot10^{-9}$	$6.27\cdot10^{-11}$	$5.51\cdot10^{4}$	$3.46\cdot10^{2}$	$1.32\cdot10^{-4}$	$3.36\cdot10^{-6}$	0.9938
		Og XI-2	$2.63\cdot10^{-9}$	$5.88\cdot10^{-11}$	$6.17\cdot10^{4}$	$3.93\cdot10^{2}$	$1.62\cdot10^{-4}$	$3.48\cdot10^{-6}$	0.9962
KUN	V_HH17 a-KUN	KUN-1	$4.05\cdot10^{-11}$	$4.13\cdot10^{-10}$	$2.08\cdot10^{4}$	$1.89\cdot10^{2}$	$8.43\cdot10^{-7}$	$8.60\cdot10^{-6}$	0.9531
		KUN-2	$<1.0\cdot10^{-12}$	$2.66\cdot10^{-11}$	$8.69\cdot10^{4}$	$3.99\cdot10^{2}$	$<1.0\cdot10^{-7}$	-	0.998
	V_HH18 a-KUN	KUN-1	$2.07\cdot10^{-9}$	$5.39\cdot10^{-11}$	$7.48\cdot10^{4}$	$5.70\cdot10^{2}$	$1.55\cdot10^{-4}$	$3.85\cdot10^{-6}$	0.9875
	V_HH19 a-KUN	KUN-1	$<1.0\cdot10^{-12}$	$3.51\cdot10^{-11}$	$1.37\cdot10^{5}$	$1.53\cdot10^{3}$	$<1.0\cdot10^{-7}$	-	0.9887
		KUN-2	$1.48\cdot10^{-10}$	$1.75\cdot10^{-11}$	$1.30\cdot10^{5}$	$6.44\cdot10^{2}$	$1.92\cdot10^{-5}$	$2.26\cdot10^{-6}$	0.9963

Supplementary Table 6. LD_{50}s for venom fractions, toxins, and venoms using the s.c. and i.v. injection routes.

	**Sample**	**LD_{50} (µg/mouse)**	
		**i.v.**	**s.c.**
	lNTx-7	1.3 [-]	ND
	sNTx-3	1.3 [1.3 to 1.4]	ND
Dendroaspis	*D. angusticeps*	38.6 [-]	41.6 [37.9 to 45.2]
	D. jamesoni	20.1 [-]	21.6 [20.2 to 22.9]
	D. polylepis	9.2 [-]	22.2 [19.1 to 25.4]
	D. viridis	9.8 [-]	12.9 [-]
Hemachatus	*H. haemachatus*	24.3 [-]	35.0 [20.4 to 24.1]
Naja (Afronaja)	*N. ashei*	16.7 [-]	ND
	N. katiensis	23.2 [-]	ND
	N. mossambica	22.1 [17.9 to 25.2]	ND
	N. nigricincta	16.5 [-]	ND
	N. nigricollis	17.1 [-]	79.3 [66.6 to 87.4]
	N. nubiae	8.7 [8.2 to 9.3]	9.6 [9.5 to 9.7]
	N. pallida	15.1 [-]	ND
Naja (Boulengerina)	*N. melanoleuca*	19.9 [-]	41.8 [-]
Naja (Uraeus)	*N. anchietae*	76.5 [72.3 to 80.6]	>200
	N. annulifera	119.2 [105.8 to 113.5]	122.6 [117.2 to 128.0]
	N. haje	10.1 [-]	11.9 [-]
	N. nivea	54.8 [54.1 to 55.5]	56.6 [53.8 to 59.2]
	N. senegalensis	10.1 [-]	12.2 [11.3 to 13.0]

Numbers in brackets [] represent 95% confidence intervals. [-] The confidence interval was undefined.

ND. LD_{50} was not determined

i.v. intravenous

s.c. subcutaneous

Supplementary Table 7. In vivo experiments for the design of the recombinant antivenom

Mouse strain	Venom/toxin	V_HHs	Molar ratio	Amount (µg)	Time of death (h)				
					M1	M2	M3	M4	M5
CD1	sNTx-3	V_HH7 a-sNTx	1:1	11.0	>24	>24	>24	-	-
CD1		V_HH5 a-sNTx	1:1	11.3	>24	>24	>24	-	-
CD1		V_HH6 a-sNTx	1:1	11.4	12	13	>24	-	-
CD1	lNTx-7	V_HH9 a-lNTx	1:1	9.9	8	19	>24	-	-
CD1		V_HH9 a-lNTx	1:2.5	24.8	>24	>24	>24	-	-
CD1	*N. haje*	V_HH9 a-lNTx	1:10	130.8	>24	>24	>24	-	-
		V_HH5 a-sNTx	1:10	134.8					
CD1	*N. melanoleuca*	V_HH9 a-lNTx	1:10	286.2	>24	>24	>24	-	-
		V_HH5 a-sNTx	1:10	185.5					
CD1	*D. viridis*	V_HH9 a-lNTx	1:10	293.2	0,3	>24	>24	-	-
		V_HH5 a-sNTx	1:10	224.9					
		V_HH15 a-Og XI	1:3	43.0					
CD1	*D. viridis*	V_HH9 a-lNTx	1:10	293.2	>24	>24	>24	-	-
		V_HH5 a-sNTx	1:10	224.9					
		V_HH15 a-Og XI	1:3	43.0					
		V_HH13 a-AgTx	1:3	39.3					
NSA	*D. viridis*	V_HH9 a-lNTx	>1:10	240.1*	>24	>24	>24	>24	>24
		V_HH5 a-sNTx	>1:10	413.1*					
		V_HH20 a-PLA_2	>1:10	291.4*					
		V_HH1 a-CTx	>1:10	746.9*					
		V_HH4 a-CTx	>1:10	746.9*					
		V_HH17 a-KUN	>1:10	479.7*					
NSA	*D. jamesoni*	V_HH9 a-lNTx	>1:10	240.1*	0,3	0,4	0,5	>24	>24
		V_HH5 a-sNTx	>1:10	413.1*					
		V_HH20 a-PLA_2	>1:10	291.4*					
		V_HH1 a-CTx	>1:10	746.9*					
		V_HH4 a-CTx	>1:10	746.9*					
		V_HH17 a-KUN	>1:10	479.7*					
NSA	*D. polylepis*	V_HH9 a-lNTx	1:10	45.4	>24	>24	>24	3 to 24	3 to 24
		V_HH5 a-sNTx	1:10	15.8					
		V_HH19 a-KUN	1:10	269.8					
		V_HH15 a-Og XI	1:10	13.8					
		V_HH13 a-AgTx	1:10	14.4					
NSA	*D. polylepis*	V_HH9 a-lNTx	1:10	45.4	>24	>24	>24	>24	>24
		V_HH5 a-sNTx	1:10	15.8					
		V_HH17 a-KUN	1:10	269.8					
		V_HH15 a-Og XI	1:10	13.8					
		V_HH13 a-AgTx	1:10	14.4					
CD1	*N. nigricollis*	V_HH20 a-PLA_2	1:10	159.9	0,3	2	>24	-	-
CD1	*N. nigricollis*	V_HH1 a-CTx	1:10	525.2	9 h	10	>24	-	-
		V_HH20 a-PLA_2	1:10	159.9					
NSA	*N. nigricollis*	V_HH1 a-CTx	1:5	262.6	>24	>24	>24	-	-
		V_HH4 a-CTx	1:5	262.6					
		V_HH20 a-PLA_2	1:10	159.9					

All experiments were performed by pre-incubating 3 LD_{50} values of venom or toxin with the selected V_HHs before i.v. injection into mice.

*V_HH amount present in the recombinant antivenom.

Supplementary Table 8. Composition of the recombinant antivenom.

Toxin (sub)family	Average MW (kDa)	Venom with highest nmoles in 3 LD_{50}s	Abundance in venom (%)	Venom LD_{50} (µg/mouse)	nmoles in 3 LD_{50}s	Specific V_HH	nmoles of V_HH in mixture	MW of V_HH (Da)	V_HH conc. (mg/mL)	V_HH in mixture (µg)	Volume (µL/mouse)
CTx	8.0	*N. nigricollis*	77.7	17.1	4.39	V_HH1 a-CTx	43.9	17,063	29.9	746.93	25.0
CTx*					4.39	V_HH4 a-CTx	43.9	17,397	39.0	746.93	19.2
sNTx	8.0	*D. jamesoni*	31.3	20.1	2.43	V_HH5 a-sNTx	24.3	17,043	27.0	413.07	15.3
lNTx	8.0	*N. haje*	40.5	10.2	1.41	V_HH9 a-lNTx	14.1	17,534	29.0	240.14	8.3
AgTx	7.5	*D. viridis*	14.4	9.7	0.52	V_HH13 a-AgTx	5.2	17,336	30.0	89.18	3.0
Og XI	7.0	*D. jamesoni*	45.0	20.1	3.50	V_HH15 a-Og XI	35.0	17,069	30.0	594.6	19.8
PLA_2	14.0	*N. melanoleuca*	27.5	19.9	1.71	V_HH20 a-PLA_2	17.1	17,329	28.7	291.40	10.2
KUN	7.0	*D. polylepis*	74.5	9.2	2.82	V_HH17 a-KUN	28.2	17,849	28.8	479.66	16.7
									**Total:**	3,601.9	117.3

*A second anti-CTx V_HH was added based on initial in vivo experiments with *N. nigricollis* venom.

Supplementary Table 9. Summary of in vivo experiments to assess the recombinant antivenom's efficacy to prevent venom-induced lethality.

Mouse strain	Venom sp.	Neutralizer	AV Dose (mg)	Time of death (hours)*					Notes
				M1	M2	M3	M4	M5	
**Recombinant AV - Pre-incubation setup**									
NSA	*N. haje*	Recombinant AV	3.6	>24	>24	>24	>24	>24	
NSA	*D. polylepis*	Recombinant AV	3.6	>24	>24	>24	>24	>24	Initial lethargy, disappeared after ~2 min for all mice
NSA	*D. viridis*	Recombinant AV	3.6	>24	>24	>24	>24	>24	Closed eyes, lethargy, and excessive grooming lasting over 24 hours
NSA	*N. nigricollis*	Recombinant AV	3.6	>24	>24	>24	>24	>24	Very few signs of distress or envenomation
NSA	*N. melanoleuca*	Recombinant AV	3.6	>24	>24	>24	>24	>24	Closed eyes and excessive grooming, other behavior normal
NSA	*D. jamesoni*	Recombinant AV	3.6	>24	>24	>24	>24	>24	
NSA	*D. angusticeps*	Recombinant AV	3.6	3	3	4,5	5	6,5	
NSA	*H. haemachatus*	Recombinant AV	3.6	>24	>24	>24	>24	>24	
NSA	*N. mossambica*	Recombinant AV	3.6	>24	>24	>24	>24	>24	
NSA	*N. pallida*	Recombinant AV	3.6	>24	>24	>24	>24	>24	
NSA	*N. ashei*	Recombinant AV	3.6	>24	>24	>24	>24	>24	
NSA	*N. katiensis*	Recombinant AV	3.6	>24	>24	>24	>24	>24	
NSA	*N. nigricincta*	Recombinant AV	3.6	>24	>24	>24	>24	>24	
NSA	*N. nubiae*	Recombinant AV	3.6	>24	>24	>24	>24	>24	
NSA	*N. anchietae*	Recombinant AV	3.6	>24	>24	>24	>24	>24	
NSA	*N. annulifera*	Recombinant AV	3.6	>24	>24	>24	>24	>24	
NSA	*N. nivea*	Recombinant AV	3.6	>24	>24	>24	>24	>24	
NSA	*N. senegalensis*	Recombinant AV	3.6	>24	>24	>24	>24	>24	
**Commercial AV - Pre-incubation setup**									
CD1	*D. viridis*	Inoserp PanAfrica	2.2	1.5	5	>24	>24	>24	
CD1	*N. nigricollis*	Inoserp PanAfrica	2.2	7	>24	>24	>24	>24	
CD1	*D. polylepis*	Inoserp PanAfrica	2.2	>24	>24	>24	>24	>24	
**Recombinant AV - Rescue setup**									
CD1	-	Recombinant AV		>24	>24	>24	>24	>24	
CD1	*N. haje*	Recombinant AV	4.8	>24	>24	>24	>24	>24	
CD1	*N. melanoleuca*	Recombinant AV	8.6	12	18	>24	>24	>24	Mobility impairment and haemorrhage of the eyes.
CD1	*D. viridis*	Recombinant AV	5.4	>24	>24	>24	>24	>24	Pain and limited mobility at 24 h. Probable haemorrhage in 2/5 mice
CD1	*D. polylepis*	Recombinant AV	9.9	2.5	3	2	0.75	2.2	
CD1	*H. haemachatus*	Recombinant AV	7.2	>24	>24	>24	>24	>24	
CD1	*D. jamesoni*	Recombinant AV	4.4	0.67	1.33	0.83	0.92	1.08	
CD1	*N. annulifera*	Recombinant AV	4.6	>24	>24	>24	>24	>24	
CD1	*N. nivea*	Recombinant AV	4.2	>24	>24	>24	>24	>24	
CD1	*N. senegalensis*	Recombinant AV	4.9	>24	>24	>24	>24	>24	
CD1	*N. nubiae*	Recombinant AV	4.5	>24	>24	>24	>24	>24	
CD1	*D. angusticeps*	Recombinant AV	4.4	0.83	0.30	0.40	0.25	0.33	
**Commercial AV - Rescue Setup**									
CD1	-	Inoserp PanAfrica	5.4	>24	>24	>24	>24	>24	
CD1	*N. haje*	Inoserp PanAfrica	2.4	>24	>24	2.2	1.7	1.7	
CD1	*D. polylepis*	Inoserp PanAfrica	5.2	1.5	>24	20	7	7	
CD1	*N. melanoleuca*	Inoserp PanAfrica	4.5	11.5	2	7.5	2.2	7.5	
CD1	*N. melanoleuca*	Inoserp PanAfrica	9.1	>24	>24	>24	>24	>24	One mouse showed severe tissue damage at the injection site
CD1	*H. haemachatus*	Inoserp PanAfrica	3.4	>24	>24	0.8	1.4	2.5	
CD1	*D. viridis*	Inoserp PanAfrica	2.8	3.8	5	12	12	24	
**$3LD_{50}$ Venom Control (i.v.)**									
CD1	*D. polylepis*	-	-	0.5 - 0.7	0.5 - 0.7	0.5 - 0.7	-	-	
NSA	*D. polylepis*	-	-	0.18	0.22	0.28	0.33	0.42	
NSA	*D. viridis*	-	-	0.42	0.75	0.15	0.16	0.28	
NSA	*N. nigricollis*	-	-	0.15	0.15	0.14	0.16	0.29	
NSA	*N. melanoleuca*	-	-	0.05	0.05	0.2	0.22	0.25	
NSA	*N. haje*	-	-	0.08	0.14	0.17	0.15	0.19	
NSA	*H. haemachatus*	-	-	0.08	0.08	0.08	0.13	0.17	
NSA	*D. angusticeps*	-	-	0.05	0.05	0.08	-	-	
NSA	*D. jamesoni*	-	-	0.05	0.05	0.03	0.07	0.1	
NSA	*N. pallida*	-	-	<0.08	<0.08	<0.08	<0.08	<0.08	
NSA	*N. mossambica*	-	-	<0.08	<0.08	<0.08	<0.08	<0.08	
NSA	*N. senegalensis*	-	-	<0.08	<0.08	<0.08	<0.08	<0.08	
NSA	*M. katiensis*	-	-	<0.08	<0.08	<0.08	<0.08	0.13	
NSA	*N. ashei*	-	-	<0.08	<0.08	<0.08	<0.08	0.25	
NSA	*N. nubiae*	-	-	0.32	0.32	0.33	0.35	0.38	
NSA	*N. nigricincta*	-	-	0.33	0.45	0.58	0.62	2.2	
NSA	*N. anchietae*	-	-	<0.05	<0.05	<0.05	<0.05	<0.05	
NSA	*N. nivea*	-	-	<0.05	<0.05	<0.05	<0.05	<0.05	
NSA	*N. annulifera*	-	-	<0.05	<0.05	<0.05	<0.05	<0.05	
**$3LD_{50}$ Venom Control (s.c.)**									
CD1	*D. polylepis*	-	-	0.35	0.38	0.46	0.46	0.46	
CD1	*N. melanoleuca*	-	-	0.78	0.68	1.27	1.1	0.77	
CD1	*N. haje*	-	-	0.7 - 0.8	0.7 - 0.8	0.7 - 0.8	0.7 - 0.8	0.7 - 0.8	
CD1	*H. haemachatus*	-	-	0.8 - 1.0	0.8 - 1.0	0.8 - 1.0	0.8 - 1.0	0.8 - 1.0	
CD1	*D. viridis*	-	-	0.43	0.83	0.85	0.48	0.65	
CD1	*D. jamesoni*	-	-	0.75	0.33	0.25	0.28	0.35	
CD1	*N. annulifera*	-	-	1.42	1.5	1.58	2,00	1.5	
CD1	*N. nivea*	-	-	1.5	1.5	1.5	1.5	1.5	
CD1	*N. senegalensis*	-	-	1.33	1.33	1.33	2.33	2.33	
CD1	*N. nubiae*	-	-	1.25	1.25	1.25	1.25	1.25	
CD1	*D. angusticeps*	-	-	0.58	0.25	0.22	0.23	0.33	

*M1 to M5 represent an individual mouse in each experiment

Note: The venom dose was 3 LD_{50}s

Supplementary Table 10. Data collection and refinement statistics V_HH1 a-CTx and Cardiotoxin complex. Statistics for the highest-resolution shell are shown in parentheses.

	**V_HH1 a-CTx - Cardiotoxin**
**Wavelength (Å)**	0.9762
**Resolution range (Å)**	51.26 - 1.6 (1.657 - 1.6)
**Space group**	P 2 2 21
**Unit cell a, b, c (Å), α, β, γ (°)**	41.94 54.90 143.01 90 90 90
**Total reflections**	339511 (41042)
**Unique reflections**	42923 (6553)
**Multiplicity**	7.9 (6.3)
**Completeness (%)**	95.87 (89.60)
**Mean I/sigma(I)**	9.49 (2.47)
**Wilson B-factor**	16.64
**R-merge**	0.13 (0.90)
**R-meas**	0.14 (0.98)
**R-pim**	0.05 (0.39)
**CC1/2**	0.996 (0.81)
**Reflections used in refinement**	42733 (3918)
**Reflections used for R-free**	2134 (195)
**R-work**	0.2220 (0.3172)
**R-free**	0.2578 (0.3480)
**Number of non-hydrogen atoms**	3076
**Macromolecules**	2782
**Ligands**	42
**Solvent**	270
**Protein residues**	356
**R.m.s.d., bond lengths (Å)**	0.008
**R.m.s.d., angles (°)**	1.13
**Ramachandran favored (%)**	97.99
**Ramachandran allowed (%)**	2.01
**Ramachandran outliers (%)**	0.00
**Rotamer outliers (%)**	0.66
**Clashscore**	8.50
**Average B-factor (Å^2)**	20.98
**Macromolecules**	20.25
**Solvent**	32.80

Supplementary Table 11. Data collection and refinement statistics V_HH5 a-sNTx and sNTx1 complex. Statistics for the highest-resolution shell are shown in parentheses.

	**V_HH5 a-sNTx – sNTx1**
**Wavelength (Å)**	0.72931
**Resolution range (Å)**	23.29 - 2.5 (2.589 - 2.5)
**Space group**	C 2 2 21
**Unit cell a, b, c (Å), α, β, γ (°)**	104.15 104.09 79.34 90 90 90
**Total reflections**	206365 (33854)
**Unique reflections**	28844 (4687)
**Multiplicity**	7.15 (7.22)
**Completeness (%)**	99.9 (100.00)
**Mean I/sigma(I)**	19.86 (3.67)
**Wilson B-factor**	62.89
**R-merge**	0.056 (0.498)
**R-meas**	0.06 (0.537)
**R-pim**	0.02 (0.20)
**CC1/2**	0.999 (0.929)
**Reflections used in refinement**	15225 (1502)
**Reflections used for R-free**	760 (75)
**R-work**	0.3238 (0.4378)
**R-free**	0.3615 (0.5450)
**Number of non-hydrogen atoms**	2903
**Macromolecules**	2852
**Ligands**	16
**Solvent**	35
**Protein residues**	376
**R.m.s.d., bond lengths (Å)**	0.005
**R.m.s.d., angles (°)**	0.90
**Ramachandran favored (%)**	86.68
**Ramachandran allowed (%)**	13.32
**Ramachandran outliers (%)**	0.00
**Rotamer outliers (%)**	0.62
**Clashscore**	12.87
**Average B-factor (Å^2)**	71.01
**Macromolecules**	70.94
**Solvent**	81.85
